# Age, sex, and temperature shape off-territory feeder use in black-capped chickadees

**DOI:** 10.1093/beheco/arae080

**Published:** 2024-10-03

**Authors:** Megan LaRocque, Jan J Wijmenga, Kimberley J Mathot

**Affiliations:** Department of Biological Sciences, University of Alberta, Edmonton, AB T6G 2E9, Canada; Department of Biological Sciences, University of Alberta, Edmonton, AB T6G 2E9, Canada; Department of Biological Sciences, University of Alberta, Edmonton, AB T6G 2E9, Canada; Canada Research Chair in Integrative Ecology, Department of Biological Sciences, University of Alberta, Edmonton, AB T6G 2E9, Canada

**Keywords:** black-capped chickadees, dominance hierarchies, foraging behavior, *Poecile atricapillus*, spatial ecology, spatial personality

## Abstract

Spatial ecology tends to focus on average movement patterns within animal groups; however, recent studies highlight the value of considering movement decisions both within and among individuals. We used a marked population of black-capped chickadees (*Poecile atricapillus*), to assess the causes and consequences of within- and among-individual differences in chickadee space use. Individuals that used feeders in addition to their most-visited “core feeder” were defined as engaging in off-territory feeder use. We found that females were more likely than males to visit off-territory feeders as ambient temperatures decrease and energetic demands increase. This may be due to sex-related differences in dominance, where males which are the dominant sex in chickadees, have priority access to feeders, while females must increase their foraging areas to meet higher energetic demand. We also found that independent of temperature, adult males were less likely than juvenile males and both juvenile and adult females to forage off-territory. We suggest that this may be due to age-specific benefits of space use in males, where un-paired juvenile males may increase feeder exploration to gain information about potential mates. Finally, we found that chickadees that had a higher propensity to visit off-territory feeders were less likely to survive to the next fall. Overall, our results suggest that dominance hierarchies and individual energetics impact within- and among-individual variation in off-territory feeder use. We provide suggestions for future studies to further investigate fitness-related consequences of within- and among-individual variation in space use.

## Teaser

Black-capped chickadees increase their daily feeder visits as ambient temperatures decrease and energetic demands increase. Females increase their visits to off-territory feeders during colder temperatures, while males maintain stable feeder use, consistent with males, the dominant sex, having priority access to feeders. Across temperatures, only males exhibited age-related differences in probability of off-territory use. We suggest juvenile males may gain additional benefits, such as prospecting potential mates, from off-territory feeder use compared to adult males.

## Introduction

Animal movement and spatial behavior are key components of population- and individual-level ecological patterns and can have important fitness outcomes (Holyoak et al. 2008; Nathan et al. 2008; Kays et al. 2015). Movement allows individuals to meet particular goals (e.g. finding a food patch), mitigate risks (e.g. evade predators), and respond to changing conditions. For example, climate change has dramatically altered temporal and spatial patterns of migration in bird populations (e.g. Neumann et al. 2022; Horton et al. 2023) and habitat fragmentation has been shown to alter the movement patterns of animal groups (e.g. Diffendorfer et al. 1995; Collinge 2000; Kerth and Melber 2009; Anadón et al. 2012; Janin et al. 2012; Poessel et al. 2014; Biddlecombe et al. 2021; Rus et al. 2021).

There is increasing evidence that individuals within animal populations consistently differ in their spatial behaviors (reviewed in Stuber et al. 2022) which is in line with previously proposed theories of animal space and habitat use, such as the Ideal Despotic Distribution (IDD; Fretwell and Lucas 1969). The IDD assumes that not all individuals have equal access to resources when occupying space within an environment and predicts that dominant individuals within a population will be able to consistently occupy and monopolize high quality territory due to their competitive advantage over subordinate individuals. As a result, subordinates are displaced and forced to use a larger, lower quality, area of space. Indeed, among-individual differences in space use behaviors such as on- versus off-territory use, vary in relation to dominance, as predicted by the IDD, such that subordinate individuals are forced off the territories monopolized by dominant individuals (e.g. Calsbeek and Sinervo 2002; Purchase and Hutchings 2008; Church and Grant 2019b, 2019a; Rohwer et al. 2020).

Individuals may differ not only in their average space use (i.e. among-individual differences) but also in their response to environmental change (i.e. plasticity or within-individual differences, also referred to as “behavioral reaction norms”) (Dingemanse et al. 2010). Importantly, among- and within-individual differences in spatial behavior may interact such that among-individual differences in space use predict within-individual plasticity in space use. For example, individuals that have relatively higher space use on average are more likely to reduce their space use as the density of territorial individuals in the population decreases (e.g. Newton and Rothery 2001; Penteriani et al. 2011; Lenda et al. 2012; Robles and Ciudad 2017). This is thought to reflect the transition from non-territoriality to territoriality when territory vacancies arise due to mortality of territorial individuals (Newton and Rothery 2001; Penteriani et al. 2011; Lenda et al. 2012; Robles and Ciudad 2017). Furthermore, among-individual differences in dominance can simultaneously influence within- and among-individual patterns of space use. For example, in a population of willow tits (*Parus montanus*), during mild winter temperatures, dominant adults forage in the innermost parts of trees more often than subordinate juveniles (Brotons et al. 2000). Under colder temperatures, dominants can achieve their required intake while maintaining their positions in the innermost parts of trees, while subordinate juveniles are forced to increase their relative use of the outer parts of trees. This example is consistent with numerous studies that have shown that when a change in environmental conditions puts stress on a population, dominant individuals are able to maintain their spatial patterns on-territory due to their competitive advantage over subordinates to control essential survival resources, while subordinates are forced to alter their patterns of space use, often expanding to use off-territory space (e.g. Desrochers et al. 1988; Hogstad 2015; Matthews and Wong 2015; Found and St. Clair 2016).

Individual differences in space use behaviors can have important fitness consequences. Spatial distribution models, such as the IDD, predict that individuals who are able to monopolize small areas of high-quality habitat will achieve a fitness advantage over those individuals that are forced to move across larger areas of low quality habitat (Fretwell and Lucas 1969). In addition, individual differences in plasticity in space use may influence individual fitness. For example, an individual that increases its space use may increase its access to both food (Sells et al. 2022) and social partners (Brown and Orians 1970), benefitting its individual fitness. However, there is also evidence that increasing space use increases both pathogen transmission (Barber and Dingemanse 2010; Boyer et al. 2010) and predation risk (Lima and Dill 1990). Thus, the fitness consequences of among-individual differences in spatial behavior are likely to be both species- and context-specific.

Avian systems are excellent models for studying spatial ecology because they exhibit a diversity of spatial behaviors, including migration (e.g. Bruderer et al. 2018), territoriality (e.g. Campioni et al. 2013), and floating (i.e. non-territoriality; e.g. Smith 1984). The spatial behavior of small non-migratory birds is particularly interesting from an energy management perspective because the winter months can be challenging due to shortened daylength and low natural food availability combined with increased costs of thermoregulation (Cooper 2000; Studd et al. 2021; Sutton et al. 2021). In addition, individual movement both consumes energy reserves and produces metabolic heat (Cooper and Sonsthagen 2007; Humphries and Careau 2011). Not surprisingly, wintering birds adjust their movement choices based on environmental changes as well as changes in habitat gaps and boundaries (Desrochers and Fortin 2000; Turcotte and Desrochers 2005; Bailey et al. 2018), food availability (Smith and Van Buskirk 1988; Brotons and Herrando 2003; Mady et al. 2021), season (e.g. breeding vs. non-breeding) (Brittingham and Temple 1992; Lemmon et al. 1997), and temperature (Alatalo 1982; Hogstad 2015).

Given the increased energetic costs of maintaining homeostasis for small over-wintering birds, decreasing winter temperature may have important implications for individual movement decisions related to gathering food. If birds have a fixed energy budget, increased costs of thermoregulation during the cold would require reduced energy expenditure on activity (Grubb 1978; Cooper 2000). Alternatively, if birds can modify their total energy budget (Cooper and Sonsthagen 2007), or allocate heat generated via activity toward thermoregulation (Humphries and Careau 2011), then higher costs of thermoregulation may be met by increasing foraging activity (e.g. Kessel 1976; Bonter et al. 2013; Latimer et al. 2018). Among-individual differences in spatial behavior have also been found to correlate with individual state variables relating to dominance rank. For example, in some avian species, older birds (Brotons et al. 2000) as well as males (Hogstad 2015) monopolize resources, allowing them to restrict their space use to smaller, safer territory areas. Therefore, analyzing possible correlates of both within- and among-individual differences in space use of small, resident winter birds may provide insight into the predictors of individual resident bird survival throughout the winter months.

Our study used a marked population of black-capped chickadees (*Poecile atricapillus*; henceforth referred to as “chickadees”) to address questions related to both within- (i.e. plasticity), and among- (i.e. personality) individual differences in space use. Our study site consists of 7 feeders that were presumed a priori to be located on unique flock territories. Because chickadees in our population had a preferred “core feeder” location (i.e. a feeder location where they fed more than twice as much as any other feeder), chickadees that only visited their “core feeder” on a given day were assumed to exhibit “on-territory” space use, while those that visited feeders other than or in addition to their “core feeder” exhibited “off-territory” space use. In this way, we were able to quantify changes in propensity to visit off-territory feeders (see also Methods). Specifically, we addressed 4 questions. (1) How is within-individual variation in space use influenced by ambient temperature? We hypothesized 2 alternative mechanisms by which chickadees might cope with increasing energetic costs of thermoregulation with decreasing ambient temperatures in winter. First, if chickadees can increase their total energy expenditure under increased costs of thermoregulation, then they would increase activity and movement behavior as a means of securing more resources, and we predicted that chickadees would increase their probability of using off-territory feeders as temperatures decrease. Alternatively, if total energy expenditure is fixed, increased costs of thermoregulation would come at the cost of other activities (such as spatial movement), and we predicted that chickadees would decrease their probability of using off-territory feeders as temperatures decrease. (2) Do individuals show repeatable variation in space use (i.e. spatial personality), and if so, are among-individual differences in space use predicted by dominance? We used age and sex as proxies for dominance rank as, in black-capped chickadees, males are dominant to females and, within sex, older birds are dominant over younger birds (Smith 1997). If dominance status determines an individual’s ability to monopolize resources within a territory, then we predicted that males would be less likely to use off-territory feeders compared with females, and within each sex, adults would be less likely to use off-territory feeders than juveniles. (3) Does dominance predict plasticity in the spatial behavior of individuals in response to decreasing ambient temperature? If dominants can monopolize feeders on-territory, we predicted that as temperature decreases, females and juveniles would have a steeper reaction norm (i.e. greater change in their space use) compared with males and adults. (4) Are among-individual differences in space use associated with differences in annual survival? We did not have strong a priori predictions for this association since our predictions depended on the results of dominance effects.

To test these predictions, we quantified within- and among-individual differences in the propensity to visit off-territory feeders. We also quantified within- and among-individual differences in daily feeder visits to evaluate the role of food acquisition in shaping space use decisions at both the within- and among-individual levels. Our results add to existing literature exploring the mechanisms underlying within- and among-individual variation in space use. We discuss potential fitness consequences of space use variation during the winter season in a non-migratory passerine and highlight important avenues for future work.

## Methods

### Study site and study population

**Fig. 1.**
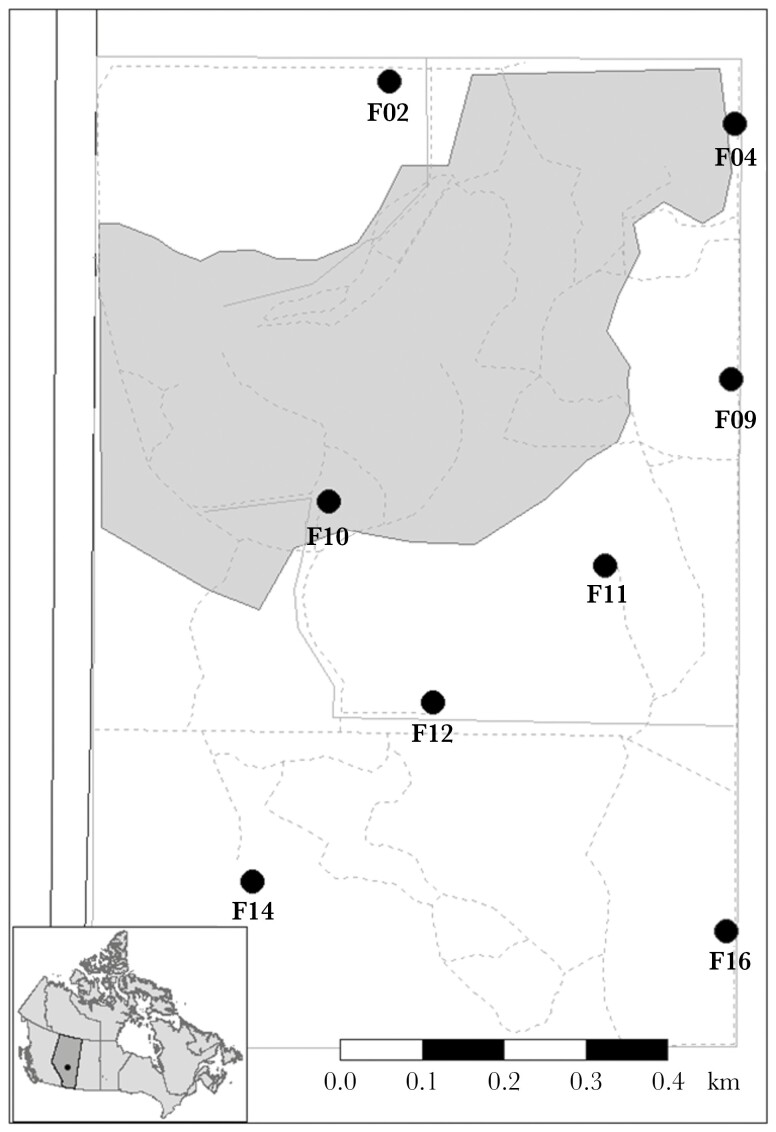
A map of the UABG. The black dot in the inset map represents the location of the field site within Canada. Garden limits are shown by solid grey lines and dotted grey lines represent walking pathways within the garden. West of the garden, the black lines represent the Devonian Highway (Alberta Highway 60), used to access the garden, and the grey shaded area represents the public visitors’ area and the managed horticulturist gardens. Feeder locations are represented by black circles and labeled with their respective numbers (02, 04, 09, 10, 11, 12, 14, and 16). Note that feeder 14 (i.e., “thermal feeder”) was removed from this study’s dataset (see Supplementary Text S2 for further details). All feeders are greater than 270m apart corresponding to published chickadee flock territory sizes (Smith 1992). For reference, the distance between feeder 04 and feeder 09 is 302m. The map was constructed by Josue Arteaga-Torres (used with permission) with feeder labels added by Megan LaRocque.

This study was conducted between October 2022 and March 2023 in a marked population of black-capped chickadees at the University of Alberta Botanic Garden (UABG) in Devon, Alberta, Canada (53°2402700 N, 113°4504100 W). The UABG is located 22 km SW of Edmonton and 6 km N of Devon within the Devon Dunes natural area. It is a 0.97 km^2^ property with 0.32 km^2^ of display gardens and 0.65 km^2^ of mixed wood forest. The marked population was established in October 2017, and standardized catching effort is done each fall (generally between October and December) to mark new birds. Birds are caught using mist nets set up near 8 feeder locations spread throughout the 0.65 km^2^ study area (see Fig. 1). Capture effort for the study year occurred between 12 November 2022, and 2 January 2023 (inclusive). Mean daily temperature was obtained from the Edmonton International Airport (YEG) weather station, located 10 km SE of the study site (data provided by Alberta Agriculture and Forestry, ACIS: https://agriculture.alberta.ca/acis).

Upon initial capture, birds are fitted with a unique metal band provided by the Canadian Wildlife Service, and a unique combination of color bands, including leg bands embedded with passive integrated transponder (PIT) tags. A small blood sample is collected to allow for molecular sexing (Griffiths et al. 1998). For birds without molecular sex data, we use a discriminant function to assign a highly probable sex (Sridharan 2021; see Supplementary Text S1). After birds are captured (whether initially or upon recapture), standard morphometric data are collected (body mass, wing length, bill length and depth, tarsus length), and the age of the bird is estimated using plumage characteristics. During fall catching, birds can be scored as hatch year or after hatch year. The birds present in our study ranged in minimum age from 0 years (i.e. hatched in spring 2022) to 6 yr (i.e. hatched in spring 2016 or earlier).

### Ethical note

This study was conducted in accordance with the University of Alberta Biosciences Animal Care and Use Committee (AUP00002210), the Alberta Wildlife Research Permit (#56631) and Collection License (#56632) and Environmental Canada Canadian Wildlife Service (banding permits #10936 and 10936A). To minimize stress during capture, we did not attempt catching during inclement weather, and mist nets were monitored continuously to ensure birds were removed from the nets and processed quickly. Any birds that appeared stressed or unwell, were released immediately without being processed. For birds that were processed, morphometric measurements, banding, and blood sampling was completed in under 10 min before birds were released at the site of capture. For molecular sexing, a small (< 20 μL) blood sample was collected from the brachial vein of initially captured birds (i.e. only one blood sample was collected for each bird).

### Spatial use data

Each feeder is equipped with an RFID antenna that automatically records the date, time, and unique 10-digit hexadecimal code of each PIT tagged individual whenever it visits a feeder. During this study year, 1 of the 8 feeders had a different antenna frequency system, thus we removed it from analyses (see Supplementary Text S2 for further details). Each of the remaining 7 feeders were filled with black-oil sunflower seeds from 29 October 2022 to 28 February 2023 (inclusive); however, we used only a subset of the data for our analyses of spatial behavior. First, we restricted the dataset based on dates and included only feeder visit data collected between 9 January 2023 and 14 February 2023. We used 9 January 2023 (1 wk post-catching) as the start date to reduce the effect that catching effort may have on spatial behavior (e.g. displacing individuals from feeders where catching was occurring). Between 15 February 2023 and 23 February 2023 (inclusive), 1 of the 7 feeders had a damaged circuit board resulting in complete loss of data during that time interval, and thus we removed this 8-d period from our data analysis. Although we did collect an additional 5 d of data at feeders from February 24th to 28th, 2023 (inclusive), we chose not to include these dates in our analyses due to (1) the large temporal break in otherwise continuous data and (2) because this break coincided with a time where the spatial dynamics of chickadee flocks were likely changing (see Fig. S1). Literature suggests that chickadees can begin to establish breeding territories in early February which can initiate winter flock break up (Smith 1992). However, we present results in the Supplementary Material that include feeder data collected from February 24th to 28th for full transparency (see Supplementary Text S3 for details and Tables S1–S3 for model results and pairwise comparisons).

We also removed individuals (*N* = 3) for which we did not have molecular sex data and additionally whose sex assignment using the discriminant function was inconclusive (see Supplementary Text S1 and Sridharan 2021). This resulted in a total of 138 uniquely identified (i.e. PIT-tagged) individuals for which we analyzed foraging activity at the 7 feeders included in this study (see Fig. 1).

We analyzed territory use at the level of days by summing the total number of unique feeders visited by each individual during each of the 37 study days from 9 January 2023 to 14 February 2023 (inclusive). We also summed the total number of feeder visits made by each individual during each of the 37 d to allow us to assess total daily feeder visits. Extensive video observations in our study population have been conducted to confirm that chickadees take a single sunflower seed per visit (Jan Wijmenga, unpublished data). Only rarely (< 1% of visits), were chickadees displaced without having taken a seed. Thus, the count of visits to the feeder strongly correlates with the number of seeds taken from the feeder. For individuals that were not detected at any feeders within a day, we assigned them a unique feeder count and daily feeder visits of “0.” If an individual was never detected at any of the feeders in any subsequent days after a unique feeder count and daily feeder visits of “0” (i.e. it had a unique feeder count and daily feeder visits of “0” from the initial ‘0’ entry until 14 February 2023), we assumed that the individual may have died, and we replaced the sequence of “0s” with “NAs.” This occurred for a total of *N* = 5 individuals.

**Fig. 2.**
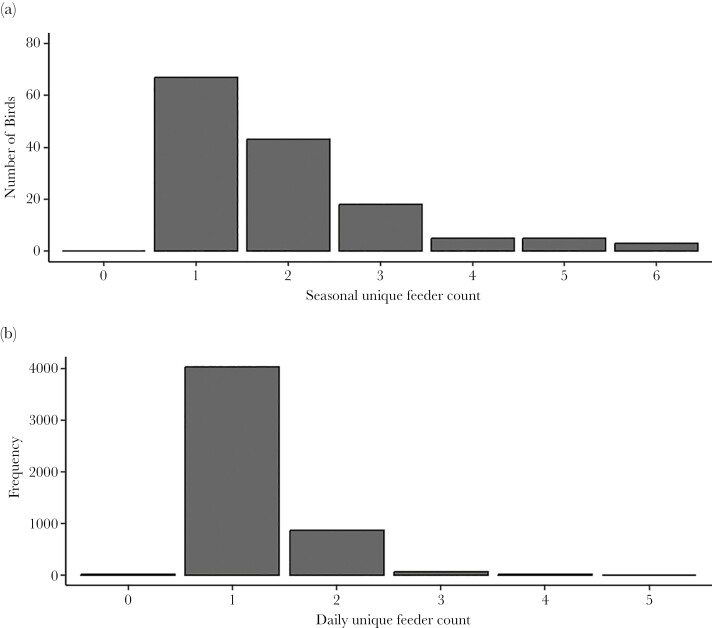
Histograms of unique feeder count for each unique bird calculated (a) across the entire study period and (b) for each day within the study period.

Initially, we planned to use unique feeder count as our proxy for territory use. However, the distribution of unique feeder counts was highly left skewed, with *N* = 70 birds out of *N* = 138 (51%) using a single feeder over the entire season (Fig. 2a), and *N* = 4,033 bird-days (i.e. unique combination of bird ID and date) out of *N* = 4,969 unique bird-day combinations (81%) using a single feeder per day (Fig. 2b). This is not surprising, because the spacing of feeders in our study population (i.e. at least 270 m between neighboring feeders) was chosen based on published estimates of chickadee winter territory size such that we aimed to have a single feeder per winter flock territory (see Smith and Van Buskirk 1988 for a review; Smith 1992; see Fig. 1). Consistent with this expectation, for birds that did use > 1 feeder through the study period (*N* = 68), all but *N* = 2 birds had a strong preference for a single feeder (termed “core feeder”) across the study period (i.e. > 60% of visits throughout the study period were to that feeder relative to any other feeder in a pairwise comparison; range 60.63% to 100%). The 2 birds that did not demonstrate strong preference for a single feeder location, had a roughly equal use of 2 feeders (the same 2 feeders for both birds). These 2 feeders (F09 and F11) were adjacent (see Fig. 1), potentially indicating that both feeders fell within their territory, and thus we coded “F09 & F11” as the “core feeder” for these individuals (which resulted in *N* = 8 levels for “core feeders”). We gave each bird a daily on- or off-territory score. On any given day, birds that visited a feeder that was not their “core feeder” were defined as visiting “off-territory” (off-territory score = 1) while birds that exclusively visited their “core feeder” on a given day were defined as visiting “on-territory” (off-territory score = 0).

### Data analysis

All statistical analyses were conducted in the R-statistical environment v. 4.3.3 (R Development Core Team 2020) using the R-studio interface (R Studio Team 2020). For analyses of territory use, we first removed all unique feeder counts of NA or zero and binned the remaining values into “on-territory” (off-territory score = 0) and “off-territory” feeder use (off-territory score = 1). To explore sources of the probability of off-territory use, we constructed a generalized linear mixed-effects model (GLMM) fitted with a binomial error distribution using the “glmer” function in the “lme4” package (v. 1.1-35.3; Bates et al. 2015). To explore sources of variation in daily feeder visits, we square root transformed daily feeder visits and constructed a linear mixed-effects model (LMM) with a Gaussian error distribution using the “lmer” function. For both our GLMM and LMM, we verified model fit using the “DHARMa” package (v. 0.4.6; Hartig 2022). We constructed a number of alternative models to ensure that models presented achieved good model fit (see Supplementary Text S4). The biological interpretation of the results were the same across a number of alternative model specifications (see Tables S4 and S5). The models presented in the main text had the best model fit of all the models considered (see Figs S2–S7).

Both the GLMM and LMM included the same fixed and random effects structures, described below. Given that most birds were minimum age 0 or 1 (Age 0: *N* = 70, Age 1: *N* = 37, Age 2: *N* = 18, Age 3: *N* = 3, Age 4: *N* = 5, Age 5: *N* = 3, Age 6: *N* = 2), we binned age into 2 categories for analysis: AgeBin = 0, birds hatched in 2022, “juveniles”; AgeBin = 1, birds hatched in 2021 or earlier, “adults.” “Age-Sex” was a composite variable specifying the age (0 = juvenile or 1 = adult) and sex (male or female) of each individual, resulting in 4 levels (juvenile male, juvenile female, adult male, adult female). We included Age–Sex and the interaction between Age–Sex and temperature as fixed effects so that we would obtain estimates for each Age–Sex category, and to account for possible interacting effects of dominance on response to temperature change. Temperature was standardized prior to analyses by dividing values by 2 standard deviations (SD) so that the estimated effect of temperature reflects the effect of 1 SD change in temperature (i.e. 5.74°C), facilitating comparison with Age–Sex effects sizes (Gelman 2008). We also left-zeroed the standardized temperature values so that the model intercept estimates for each Age–Sex category would be at the lowest temperature in our dataset (i.e. −17.1°C). We used means parameterization to estimate the intercepts for each Age–Sex category separately, thus allowing for direct assessment of contrasts for all pairwise comparisons. We also included “core feeder” as a random effect in both models to account for non-independence of repeated measures from individuals sharing the same core feeder location. Finally, we included bird ID as a random effect in both models to account for non-independence of repeated measures data on the same individuals, and to allow for estimation of among-individual variance and repeatability of off-territory use (binary) and daily feeder visits (continuous). For non-Gaussian models, such as the probability of using off-territory feeders, it is common for some permutation iterations to estimate repeatability using the “rpt” function from the “rptR” package (Stoffel et al. 2017) to not converge, which was the case here. Therefore, we calculated the adjusted repeatability for off-territory spatial behavior using the latent scale repeatability equation for binomial data presented in Nakagawa and Schielzeth (2010) and set the overdispersion parameter to 1 (since our model was not over-dispersed). Adjusted repeatability for daily feeder visits was estimated using the “rpt” function in the “rptR” package (v. 0.9.22; Stoffel et al. 2017).

We obtained the fixed effect mode and 95% credible intervals (CrIs) of the posterior distribution of 1000 simulations of the models using the “sim” function of the “arm” package (v. 1.14.4; Gelman and Su 2014). We used the 95% CrI to evaluate the level of support for a given effect. 95% CrIs that did not overlap zero were described as providing strong support for an effect, while estimates that were centered on zero were described as providing strong support for lack of an effect, or no support for an effect. For estimates not centered on zero but whose 95% CrI overlapped zero, we calculated the proportion of estimates that were above (for negative mean estimates) or below (for positive mean estimates) zero (i.e. proportion overlap, or pr), to aid in the interpretation of the strength of support. We interpreted estimates biased away from zero but whose CrIs had up to 15% overlap with zero (i.e. pr ≤ 0.15) as providing moderate support for an effect because this corresponds to 5 times greater support (i.e. 0.75/0.15) for the interpretation of an effect in the reported direction compared with the interpretation of an effect in the opposing direction (Marsman and Wagenmakers 2017).

Because pairwise overlapping of confidence intervals does not necessarily mean that the 2 estimates are the same, we also used 95% CrIs to evaluate the level of support for differences in pairwise comparisons of estimates (Greenland et al. 2016). Pairwise CrIs that did not overlap each other were described as providing strong support for a difference between the estimates. For pairwise CrIs that overlapped with each other, we calculated the difference between each estimate resulting from the simulation and calculated the proportion of estimate differences that were above (for negative differences) or below (for positive differences) zero, to aid in the interpretation of the strength of support. We interpreted estimate differences in the same way as described above (Cumming and Finch 2005; Marsman and Wagenmakers 2017).

**Fig. 3.**
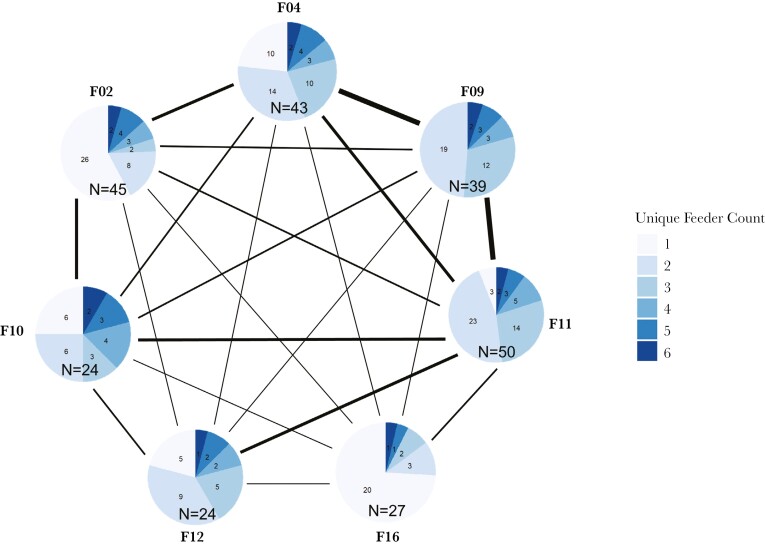
The number of unique birds that visited each feeder and a representation of pairwise feeder connectedness. Each feeder’s pie chart (labeled with their respective numbers) shows the number of unique birds which visited the focal feeder (i.e. unique feeder count = 1) and other feeders (unique feeder count > 1). The number of unique birds (*N*) which visited each feeder at least once throughout the study period is indicated at the bottom of each pie chart. A line connecting a pair of feeders represents birds that visited both feeders throughout the study period. Line width represents the number of unique individuals that visited each feeder pair (1pt line represents *N* = 1, 2, 3, 4; 2pt line represents *N* = 7, 9; 3.5pt line represents *N* = 13, 14, 16; 6pt line represents *N* = 26, 27).

Given that “core feeder” accounted for substantial variance in the probability of using off-territory feeders (see Results and Table 1), we conducted post-hoc checks to confirm that spatial behavior patterns were not driven by “core feeder”-related differences in opportunities to discover or visit off-territory feeders. For example, it would be conceivable that distance between “core feeder” and adjacent feeders determined opportunities to discover and use feeders. Although no birds visited all 7 feeders throughout the study period (range: 1 to 6, Fig. 2a), all pairwise combinations of feeders were used, including the 2 feeders located at the greatest distance to each other (F02 and F16) (see Fig. 3). Thus, we conclude that birds could conceivably visit any feeder in the study area and were not limited in their ability to visit all feeders based on physical proximity of feeders. We also conducted rarefaction analyses to address the possibility that certain “core feeders” disproportionately drove the patterns reported in Table 1. We excluded each feeder location (F02, F04, F09, F10, F11, F12, F16) one-by-one and re-ran the analyses described above, resulting in a total of 7 rarefactions (see Supplementary Text S5 for more details). We found that regardless of the feeder location that was excluded, off-territory use and daily feeder visit results were quantitively similar (see Tables S8–S11 for model results and pairwise comparisons).

**Table 1.** Model results for probability of foraging off-territory (binomial GLMM) and total daily feeder visits (square root transformed, LMM) as a function of Age-Sex and temperature (temperature effect calculated for each Age-Sex category).

	Log odds (foraging off-territory/foraging on-territory)	Square root (daily feeder visits)
Fixed effects	*β (95% CrI)* *proportion overlap (pr)*	β (95% CrI)
Female—Juvenile*	−1.99 (−4.74, −0.15)	8.50 (8.10, 9.12)
Female—Adult*	−2.84 (−4.85, 0.17) *pr = 0.04*	8.48 (7.78, 8.90)
Male—Juvenile*	−2.26 (−4.55, 0.11) *pr = 0.03*	9.73 (9.03, 10.12)
Male—Adult*	−4.40 (−6.26, −1.37)	9.44 (8.87, 9.93)
Female—Juvenile: Temperature	−0.57 (−0.92, 0.01) *pr = 0.03*	-1.10 (-1.24, -0.88)
Female—Adult: Temperature	−0.78 (−1.28, −0.23)	-0.78 (-1.02, -0.61)
Male—Juvenile: Temperature	0.23 (−0.33, 0.57) *pr = 0.28*	-1.49 (-1.68, -1.32)
Male—Adult: Temperature	0.02 (-0.41, 0.60) *pr = 0.33*	-1.30 (-1.44, -1.07)
Random Effects	σ (95% CrI)	σ (95% CrI)
Bird ID *N* = 138	4.43 (3.76, 5.72)	2.09 (1.88, 2.32)
Core Feeder ID *N* = 8	11.70 (2.52, 24.09)	0.05 (0.03, 0.09)
Residual *N* = 4,969	1 (--)†	1.86 (1.80, 1.94)
Repeatability	r (95% CrI)	r (95% CrI)
Bird ID *N* = 138	0.57 (0.53, 0.63)	0.51 (0.43, 0.57)

Units for probability of foraging off-territory slope estimates are changes in the log-odds ratios (off-territory use: on-territory feeder use) per 1 SD increase in temperature. Units for feeder visit rate slope estimates are changes in the square root of the total number of daily feeder visits per 1 SD increase in temperature. Proportion overlap (pr) values are reported for estimates with CrIs that overlap zero (see Methods section for more details). *Intercept values for Age–Sex categories are estimated at −17.1°C (the lowest temperature in our dataset), and temperature was standardized prior to analysis, therefore estimate effect sizes are for 1 SD change in temperature (i.e. 5.74°C). †Residual variance is fixed to one for binary traits

Next, we evaluated whether among-individual differences in daily feeder visits and probability of feeding off-territory were associated with differences in annual survival. To do this, we obtained RFID detection data for the birds included in our study in the subsequent fall (10 September 2023, through 21 October 2023). Juvenile chickadees disperse from their natal family groups in the summer and by late fall, flocks of unrelated individuals (i.e. previously mated pairs and new juveniles) are formed (Odum 1942; Smith 1976, 1992, 1994; Weise and Meyer 1979). Thus, by the time our data were collected, chickadees had already formed stable winter flocks that should persist throughout the winter and across years. Therefore, we assumed that birds which were not detected the following fall had died rather than emigrated and were assigned a survival value of 0 (*N* = 58). Birds that were detected were confirmed to have survived and were assigned a survival value of 1 (*N* = 80). Initially, we tried to estimate the among-individual correlation between off-territory use and survival, and between daily feeder visits and survival using 2 separate bivariate models. However, we were unable to achieve good model convergence across numerous prior specifications. Therefore, we instead followed an approach recommended by Hadfield et al. (2010). We extracted the best linear unbiased predictors (BLUPs) from the off-territory use and daily feeder visits models described above. Then, we constructed 2 separate univariate generalized linear models (GLMs) of survival (yes/no) with a binomial error distribution as a function of (1) the off-territory use BLUPs and (2) the daily feeder visits BLUPs using the “glm” function in that “lme4” package (Bates et al. 2015). Both model BLUPs were scaled using the “scale” function in R to allow for appropriate estimate comparison. The “scale” function divides each observed value by 1 SD so that estimated effect sizes are for 1 SD in BLUP values. To account for BLUP uncertainty, we ran each GLM of survival 1,000 times using an estimate drawn from the distribution of BLUPs for off-territory use and daily feeder visits. The 1000 estimated effects sizes of off-territory use and daily feeder visits on survival were fitted to derive posterior distributions for the estimated effect size and 95% CrI for the relationship between off-territory use and daily feeder visits on annual survival. We obtained log odds ratios for survival model estimates. A log odds ratio above zero indicates a survival probability above 50%, while a log odds ratio below zero indicates a survival probability of less than 50%.

The detailed structure of all models, priors, and code used for analyses are available on the Dryad Digital Repository (10.5061/dryad.47d7wm3pn; LaRocque et al. 2024), along with the full data used for analysis.

## Results

**Fig. 4.**
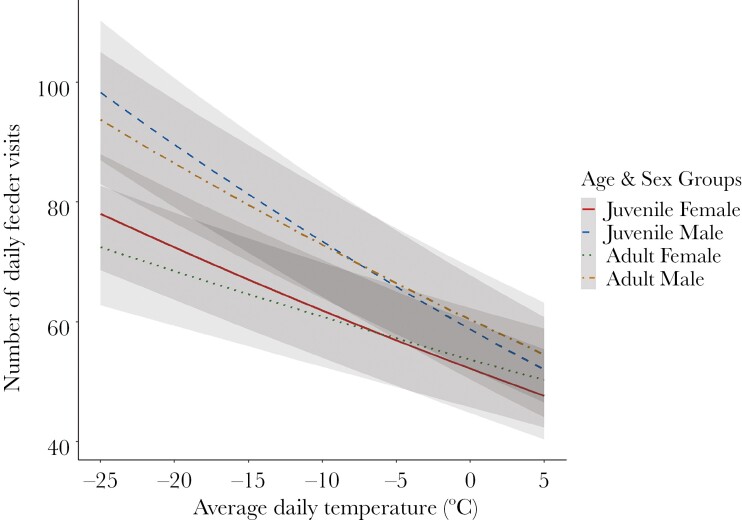
Predictions from the model for total daily feeder visits in response to the average daily temperature as a function of age (juvenile, adult) and sex (female, male). Note that the model was run with square root transformed daily feeder visits, but estimates were back transformed so that visualized values are total daily feeder visits. The lines represent regressions, and the gray regions represent 95% CrIs. (Online version in color.)

Because our predictions for spatial patterns were contingent on whether chickadees adjust their total energy expenditure in response to temperature and/or differ in energy expenditure, we first looked at the effect of temperature, sex, and age on daily feeder visits (Table 1). We found that under the coldest winter condition in our dataset (mean daily temperature = −17.1°C), juvenile males (β = 9.73, 95% CrI = 9.03, 10.12) and adult males (β = 9.44, 95% CrI = 8.87, 9.93) made more visits to feeders (square root transformed) than juvenile females (β = 8.50, 95% CrI = 8.10, 9.12) and adult females (β = 8.48, 95% CrI = 7.78, 8.90) (see Table 2 for pairwise contrasts and proportion overlap of CrIs). We also found that all Age–Sex groups showed a significant change in the square root number of daily feeder visits they made as a function of temperature (change in daily visits per 1 SD change in temperature (5.74°C); juvenile females: β = −1.10, 95% CrI = −1.24, −0.88; juvenile males: β = −1.49, 95% CrI = −1.68, −1.32; adult females: β = −0.78, 95% CrI = −1.02, −0.61; adult males: β = −1.30, 95% CrI = −1.44, −1.07), such that each Age–Sex group increased their daily feeder visits under colder conditions (Fig. 4; see Table 2 for pairwise contrasts and proportion overlap of CrIs). Finally, we found the among-individual differences in the square root number of daily feeder visits to be highly repeatable in our population (*r* = 0.51, 95% CrI = 0.43, 0.57).

**Table 2.** Pairwise contrasts between Age-Sex categories and proportion overlap values (pr) for daily feeder visit effect sizes. Estimated differences (contrasts) are calculated by subtracting row heading Age-Sex category from column heading Age-Sex category. Above the diagonal are intercept contrasts and below the diagonal are slope contrasts (i.e. temperature interaction).

	Female-Juvenile	Female-Adult	Male-Juvenile	Male-Adult
**Female-Juvenile**	----------------------------	−0.41 (−0.93, 0.47) *pr = 0.24*	0.71 (0.07, 1.47) *pr = 0.01*	1.07 (0.54, 1.95) *pr = 0.00*
**Female-Adult**	−0.23 (−0.55, −0.02) *pr = 0.02*	-----------------------------	1.12 (0.23, 1.65) *pr = 0.00*	0.98 (0.24, 1.74) *pr = 0.00*
**Male-Juvenile**	0.20 (−0.05, 0.45) *pr = 0.06*	0.69 (0.44, 0.98) *pr = 0.00*	---------------------------	−0.37 (−0.79, 0.58) *pr = 0.30*
**Male-Adult**	0.40 (0.15, 0.68) *pr = 0.00*	0.48 (0.22, 0.75) *pr = 0.00*	−0.24 (−0.47, 0.06) *pr = 0.05*	------------------------

**Fig. 5.**
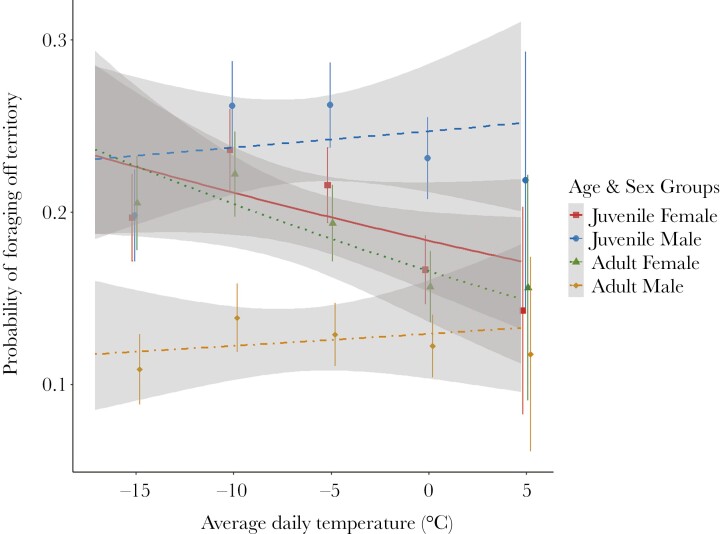
Predictions from the model for probability of off-territory feeder use in response to the average daily temperature as a function of age (juvenile, adult) and sex (female, male). The points represent the mean probability of foraging off-territory for birds of corresponding age and sex within a 5°C temperature bin calculated from raw data. Error bars represent the standard error of this mean value. Fit lines with 95% CI for each Age–Sex category were generated using the “stat_smooth” argument in the “ggplot” package (v. 3.5.0; Wickham 2016) on raw data. Note that lines are only fitted within the range of observed temperatures. The grey regions represent 95% CrIs. (Online version in color.)

The median number of feeders visited per day was 1 (range: 0 to 5) and individuals were detected on 36.01 out of 37 possible days (SD = 4.60, range: 2 to 37). Throughout the study period, *N* = 70 individuals visited exclusively 1 feeder per day, while the other *N* = 68 individuals visited more than 1 feeder per day on at least some occasions (Fig. 2a). We also observed sex and temperature related effects, with additional effects of age, on the probability of visiting off-territory feeders (Table 1). Under the coldest winter condition in our data set (−17.1°), adult males had a lower log odds of visiting off-territory feeders (β = −4.40, 95% CrI = −6.26, −1.37), than juvenile males (β = −2.26, 95% CrI = −4.55, 0.11, pr = 0.03), juvenile females (β = −1.99, 95% CrI = −4.74, −0.15), and adult females (β = −2.84, 95% CrI = −4.85, 0.17, pr = 0.04). There was strong support for a difference between adult males and all other Age−Sex groups; however, there was no support for differences between juvenile females, adult females, and juvenile males (see Table 3 for pairwise contrasts and proportion overlap of CrIs). We also found that response to temperature varied as a function of sex, but not age (Fig. 5). Specifically, as temperature increased, the log odds of visiting off-territory feeders did not change for either juvenile males (β = 0.23, 95% CrI = −0.33, 0.57, pr = 0.28) or adult males (β = 0.02, 95% CrI = −0.41, 0.60, pr = 0.33). However, there was strong support that females decreased the log odds of visiting off-territory feeders as temperature increased (juveniles: β = −0.57, 95% CrI = −0.92, 0.01, proportion overlap = 0.03; adults: β = −0.78, 95% CrI = −1.28, −0.23) (see Table 3 for pairwise contrasts and proportion overlaps). Finally, we found among-individual differences in the log odds of visiting off-territory feeders to be highly repeatable in our population (*r* = 0.57, 95% CrI = 0.53, 0.63).

**Table 3.** Pairwise contrasts between Age-Sex categories and proportion overlap (pr) values for off-territory use effect sizes. Estimated differences (contrasts) are calculated by subtracting row heading Age-Sex category from column heading Age-Sex category. Above the diagonal are intercept contrasts and below the diagonal are slope contrasts (i.e. temperature interaction).

	Female-Juvenile	Female-Adult	Male-Juvenile	Male-Adult
**Female-Juvenile**	----------------------------	−0.02 (−1.19, 1.76) *pr = 0.51*	−1.51 (−2.86, 0.14) *pr = 0.03*	−0.36 (−1.26, 1.45) *pr = 0.54*
**Female-Adult**	0.34 (−0.47, 0.94) *pr = 0.24*	-----------------------------	0.32 (−1.26, 1.45) *pr = 0.43*	−1.27 (−2.99, 0.05) *pr = 0.03*
**Male-Juvenile**	−0.61 (−1.26, 0.14) *pr = 0.05*	−0.77 (−1.62, −0.22) *pr = 0.01*	---------------------------	−1.54 (−3.12, −0.23) *pr = 0.02*
**Male-Adult**	−0.59 (−1.24, 0.01) *pr = 0.03*	−1.03 (−1.62, −0.16) *pr = 0.01*	−0.08 (−0.61, 0.75) *pr = 0.51*	------------------------

Out of the *N* = 138 individuals included in the present study, *N* = 80 birds were detected at feeders in the following Fall 2023 (57.97% survival rate). We found strong support for a negative effect of probability of off-territory feeder use on survival (β = −0.24, 95% CrI = −0.50, −0.05) and moderate support for a positive effect of daily feeder visits on survival (β = 0.12, 95% CrI = −0.02, 0.20, proportion overlap = 0.04). Low sample sizes precluded us from assessing these survival effects separately for each Age–Sex class; however, we present overall survival rates by Age-Sex class in the Supplementary Material (see Supplementary Text S6).

## Discussion

We tested the effect of ambient temperature and individual age and sex on spatial behavior and daily feeder visits in black-capped chickadees across a greater than 20°C temperature range (min: −17.1°C; max: 4.7°C). We hypothesized 2 different mechanisms by which chickadees might cope with increasing energetic costs of thermoregulation with decreasing ambient temperatures in winter. First, if chickadees can increase their total energy expenditure under increased costs of thermoregulation, we predicted they would increase activity and movement behavior as a means of securing more resources (i.e. increasing daily feeder visits). Alternatively, if total energy expenditure is fixed, we predicted no change in total daily feeder visits, and therefore that increased costs of thermoregulation would come at the cost of other activities (such as spatial movement). All chickadees, regardless of age or sex, increased daily feeder visits with decreasing ambient temperatures, consistent with the notion that chickadees increase total energy expenditure to meet the higher costs of thermoregulation. However, males, which are dominant to females (Odum 1942; Hartzler 1970; Smith 1976, 1992), achieved this without changing patterns of space use, while subordinate females increased their probability of using off-territory feeders with decreasing ambient temperatures. Taken together, our results suggest that dominance hierarchies based on age and sex as well as individual energetics play a role in shaping both among- and within-individual variation in space use as a function of temperature change in our population. Interestingly, we also observed age effects on spatial behavior in male, but not female chickadees, with juvenile males having a higher probability of using off-territory feeders compared with adult males. Off-territory feeder use in juvenile males was not different from either juvenile or adult females. This result cannot be explained solely by age- and sex-related dominance hierarchies in chickadees, as juvenile males are dominant over females (Odum 1942; Hartzler 1970; Smith 1976, 1992). We suggest that patterns of space use in males may also be shaped by age-specific differences in access to potential breeding partners, and we discuss how future studies might test this. Finally, we found that higher probability of using off-territory feeders was associated with lower annual survival, suggesting that off-territory forays impart significant fitness costs in chickadees.

We found that males make more daily feeder visits than females, regardless of age. This result is in line with other studies that found sex, but not age, effects on feeding rate in chickadees (Brittingham and Temple 1992; Wilson 2001). Our finding that males make more daily feeder visits than females may be because males are dominant to females (Odum 1942; Hartzler 1970; Smith 1976, 1992), allowing them to monopolize feeders to achieve more daily feeder visits (Ficken et al. 1990). In addition, because males are structurally larger than females, they have higher total metabolic rates compared with females (Desrochers 1989; Ramsay and Ratcliffe 2003; Lewden et al. 2012), and therefore require a higher food intake to meet energy demands.

However, we found no evidence of sex-related differences in the effect of temperature on daily feeder visits. As temperatures decreased, and therefore costs of thermoregulation increased, all chickadees increased daily feeder visits in a similar fashion, regardless of age/sex (Fig. 4). This is consistent with other studies that have assessed the effects of temperature on over-winter feeder use in chickadees (Bonter et al. 2013; Latimer et al. 2018). While the sex-specific patterns of daily feeder visits were consistent with dominance and/or whole-body metabolic rate shaping total daily feeder visits in this study, we have observed variable sex-specific patterns in daily feeder visits in our study population across years and studies. While in most cases, males have been found to have higher feeder visit rates than females (Arteaga-Torres et al. 2020; Sridharan 2021), we have observed males to have lower feeder visit rates than females in one study year (although food was not offered continuously; Haave-Audet et al. 2024), and observed no overall sex-related differences in feeder visit rate across 4 previous study years (LaRocque et al. 2023). However, each of these studies compared feeder visits between males and females within a specific feeder location, not the sum of feeder visits across all feeder sites, and thus are not directly comparable to the current study. More work is required to understand which year-specific factors shape sex-specific feeding rates in chickadees.

Given that chickadees increase their food intake to meet increased costs of thermoregulation, we were interested in understanding how this would affect patterns of space use. Specifically, we predicted that dominant birds (males) would have priority access to feeders and would therefore be able to increase total daily feeder visits without increasing use of off-territory feeders, while subordinates (females) would require a higher space use to meet their higher energy demands. As predicted, as temperature decreased, males showed no change in their propensity to visit off-territory feeders, while females increased their use of off-territory feeders (Fig. 5). This is consistent with previous work that found that male chickadees tend to have priority access to feeders and are able to competitively exclude subordinate individuals from these food resources (Ficken et al. 1990).

However, we also found that juvenile males were more likely to use off-territory feeders compared with adult males, regardless of temperature. Furthermore, off-territory feeder use by juvenile males did not differ from females of either age category (Fig. 5). This cannot be explained by dominance hierarchies alone, as juvenile males are dominant to females in chickadees (Odum 1942; Hartzler 1970; Smith 1976, 1992). One possible explanation that we suggest is that age-related differences in off-territory feeder use in males may reflect age-specific differences in the benefits of spatial exploration in males that do not exist for females. Specifically, we suggest that the higher off-territory feeder use observed in juvenile males compared with adult males may reflect a floater strategy to increase their encounter rates with available females. Juvenile males in our population are more likely to be un-paired compared with adult males by definition, because they have no prior breeding experience. As such, juvenile males may have a higher tendency to “float” between flocks, searching for opportunities to insert into higher ranking mate-pairs (Smith 1984). Although juvenile females are similarly unpaired in their first winter, they would not be expected to derive a similar benefit from off-territory exploration since in chickadees males search for females (and not vice versa) (Smith 1992). This suggests that rather than taking advantage of priority access to feeders, juvenile males may increase their use of off-territory feeders to increase their access to future mates and/or insert themselves into widowed mate-pairs. This explanation is consistent with results from earlier studies that found that subordinate male mountain chickadees (*Poecile gambeli*) explore more than dominant males (Fox et al. 2009) and male blue tits (*Cyanistes caeruleus*) that visited more social groups in the non-breeding season were more likely to acquire a mate in the breeding season (Beck et al. 2021). Future studies could address this age-specific space use by assessing whether juvenile males that have a higher propensity to forage off-territory in the winter also have higher success in finding a mate the following spring. We would also expect that if the population’s sex ratio became skewed toward a lower percentage of females than males, then juvenile males would further increase their space use in an attempt to find potential mates.

Because we found that on- versus off-territory feeder use was highly repeatable among individuals, even after considering individual sex and age differences, we predicted that among-individual variation in fitness consequences may exist. We found strong support that individuals that were more likely to use off-territory feeders were less likely to survive to the next fall. However, whether this indicates that individuals with lower survival probability are more likely to have high off-territory feeder use, or whether higher off-territory feeder use leads to lower survival, or both, cannot be teased apart with the current data. Off-territory feeder use is likely to incur both costs and benefits. One potential benefit of increasing the use of off-territory feeders is increased access to food (Sells et al. 2022). However, we found that higher probability of visiting feeders off-territory was not associated with higher total daily feeder visits across age–sex categories; adult males were able to achieve total daily feeder visits equal to or greater than all other age–sex categories while having the lowest probability of foraging off-territory. Females (both juvenile and adult) and juvenile males may have increased their daily feeder visits by visiting off-territory feeders. However, even if this benefit existed, it was not sufficient to offset putative costs of higher off-territory feeder use, such as potential increased pathogen transmission (Barber and Dingemanse 2010; Boyer et al. 2010) or increased predation risk (Lima and Dill 1990). Given that we suggest juvenile males may gain other fitness benefits from foraging off territory, such as mate prospecting opportunities, it would be important to assess whether the lower annual survival associated with higher off-territory feeder use is offset by fitness benefits of increased reproductive success in the breeding season for those males that do forage off-territory through the winter.

We also found that individuals that have higher daily feeder visits are more likely to survive to the next fall. This is consistent with a study in our population where feeder visit rate was positively correlated with annual survival (Haave-Audet et al. 2024). However, another study in our population found that feeder visit rate did not predict annual survival (Mathot et al. 2022). Indeed, there is conflicting evidence for the effect of increased food access on the survival of wild bird populations (e.g. Jansson et al. 1981; Brittingham and Temple 1988; Adelman et al. 2015; Becker et al. 2015; Broggi et al. 2021; Krama et al. 2023). Survival probability and the effects of food access may vary across years (Lahti et al. 1998). Thus, year-specific environmental conditions likely play an important role in shaping individual feeding rates, off-territory feeder use, survival, and the relationships between these traits. Although we document potentially biologically important differences in the propensity to feed off-territory in relation to age and sex in terms of survival consequences, replicating this study across years with variable environmental conditions is needed to identify whether these relationships are general or context-specific. Furthermore, there is evidence that over-winter survival is affected by both age and sex in chickadees, where more dominant individuals (i.e. males, adults) are generally more likely to survive than subordinates (i.e. females, juveniles) (Desrochers, Hannon, Nordin 1988). Because we found that off-territory feeder use and daily feeder visits were influenced by age and sex, it is possible that the observed positive correlation between feeder visits and survival and the negative correlation between off-territory use and survival at least partially reflect age and/or sex related differences in survival, independent of off-territory use and/or daily feeder visits. Unfortunately, low sample sizes for each Age–Sex category precluded us from teasing these effects apart; however, we report survival rates of each Age–Sex category in the Supplementary Material (Text S5).

Taken together, our results are consistent with literature on chickadee over-winter feeder dominance where males tend to monopolize and have priority access to food resources. However, we found evidence that adult males had the lowest probability of visiting off-territory feeders, while juvenile males and females (regardless of age) did not differ from each other. This suggests that additional factors are at play in shaping age- and sex-specific spatial patterns of feeder use. We suggest that mating history (i.e. pair-bonded or not) may shape the probability of foraging off territory in males. Specifically, juvenile males may use more space to increase their encounters with available females with which to mate in the upcoming spring. In addition, we found that higher off-territory feeder use was associated with a lower likelihood of survival, suggesting potentially important costs associated with off-territory forays. As passive movement-tracking technologies advance, it is important to consider the impact of within- and among-individual variation as well as their interaction, on individual- and population-level movement decisions (Morales et al. 2010; Spiegel et al. 2017; Hertel et al. 2020). Our study adds to the growing literature assessing how individuals within a population may differ in their spatial behaviors and how these differences may have fitness-related consequences, providing support for dominance-related spatial distribution models of populations (such as IDD). Specifically, we assessed these differences in a population that experiences exacerbated effects of cold temperatures, where movement decisions have the potential to substantially influence thermoregulatory demands (both positively and negatively). We suggest that future studies assess the impacts of winter space use on additional fitness proxies, such as reproductive success, and within- and across-season Age-Sex dominance interactions to better understand the consequences of within- and among-individual differences in space use for small, resident winter birds.

## Supplementary Material

arae080_suppl_Supplementary_Material

## Data Availability

Analyses reported in this article can be reproduced using the data provided by LaRocque et al. (2024).

## References

[CIT0001] Adelman JS , Moyers SC, Farine DR, Hawley DM. 2015. Feeder use predicts both acquisition and transmission of a contagious pathogen in a North American songbird. Proc Biol Sci. 282:20151429. 10.1098/rspb.2015.1429 26378215 PMC4614752

[CIT0002] Alatalo RV. 1982. Effects of temperature on foraging behaviour of small forest birds wintering in northern Finland. Ornis Fenn. 59:1–12.

[CIT0003] Anadón JD , Wiegand T, Giménez A. 2012. Individual-based movement models reveals sex-biased effects of landscape fragmentation on animal movement. Ecosphere. 3:1–32. 10.1890/es11-00237.1

[CIT0004] Arteaga-Torres JD , Wijmenga JJ, Mathot KJ. 2020. Visual cues of predation risk outweigh acoustic cues: a field experiment in black-capped chickadees. Proc Biol Sci. 287:20202002. 10.1098/rspb.2020.2002 33023412 PMC7657859

[CIT0005] Bailey JM , Reudink MW, LaZerte SE, Paetkau M, Johnson CJ, Hill DJ, Otter KA. 2018. Using radio frequency identification (RFID) to investigate the gap-crossing decisions of Black-capped Chickadees (*Poecile atricapillus*). Auk. 135:449–460. 10.1642/auk-17-162.1

[CIT0006] Barber I , Dingemanse NJ. 2010. Parasitism and the evolutionary ecology of animal personality. Philos Trans R Soc London Ser B. 365:4077–4088. 10.1098/rstb.2010.0182 21078659 PMC2992744

[CIT0007] Bates D , Mächler M, Bolker B, Walker S. 2015. Fitting linear mixed-effects models using lme4. J Stat Softw. 67:48. 10.18637/jss.v067.i01

[CIT0008] Beck KB , Farine DR, Kempenaers B. 2021. Social network position predicts male mating success in a small passerine. Behav Ecol. 32:856–864. 10.1093/beheco/arab034 34690546 PMC8528538

[CIT0009] Becker DJ , Streicker DG, Altizer S. 2015. Linking anthropogenic resources to wildlife–pathogen dynamics: a review and meta‐analysis. Ecol Lett. 18:483–495. 10.1111/ele.12428 25808224 PMC4403965

[CIT0010] Biddlecombe BA , Bayne EM, Lunn NJ, McGeachy D, Derocher AE. 2021. Effects of sea ice fragmentation on polar bear migratory movement in Hudson Bay. Mar Ecol Prog Ser. 666:231–241. 10.3354/meps13684

[CIT0011] Bonter DN , Zuckerberg B, Sedgwick CW, Hochachka WM. 2013. Daily foraging patterns in free-living birds: exploring the predation-starvation trade-off. Proc R Soc B. 280:20123087. 10.1098/rspb.2012.3087 PMC3652453 23595267

[CIT0012] Boyer N , Réale D, Marmet J, Pisanu B, Chapuis JL. 2010. Personality, space use and tick load in an introduced population of Siberian chipmunks *Tamias sibiricus*. J Anim Ecol. 79:538–547. 10.1111/j.1365-2656.2010.01659.x 20202009

[CIT0013] Brittingham MC , Temple SA. 1988. Impacts of supplemental feeding on survival rates of black-capped chickadees. Ecology. 69:581–589. 10.2307/1941007

[CIT0014] Brittingham MC , Temple SA. 1992. Use of winter bird feeders by black-capped chickadees. J Wildl Manag. 56:103–110. 10.2307/3808797

[CIT0015] Broggi J , Hohtola E, Koivula K. 2021. Winter feeding influences the cost of living in boreal passerines. Ibis. 163:260–267. 10.1111/ibi.12862

[CIT0016] Brotons L , Herrando S. 2003. Effect of increased food abundance near forest edges on flocking patterns of Coal Tit *Parus ater* winter groups in mountain coniferous forests. Bird Study. 50:106–111. 10.1080/00063650309461301

[CIT0017] Brotons L , Orell M, Lahti K, Koivula K. 2000. Age-related microhabitat segregation in willow tit *Parus montanus* winter flocks. Ethology. 106:993–1005. 10.1046/j.1439-0310.2000.00622.x

[CIT0018] Brown JL , Orians GH. 1970. Spacing patterns in mobile animals. Annu Rev Ecol Syst. 1:239–262. 10.1146/annurev.es.01.110170.001323

[CIT0019] Bruderer B , Peter D, Korner-Nievergelt F. 2018. Vertical distribution of bird migration between the Baltic Sea and the Sahara. J Ornithol. 159:315–336. 10.1007/s10336-017-1506-z

[CIT0020] Calsbeek R , Sinervo B. 2002. An experimental test of the ideal despotic distribution. J Anim Ecol. 71:513–523. 10.1046/j.1365-2656.2002.00619.x

[CIT0021] Campioni L , Delgado MM, Lourenço R, Bastianelli G, Fernández N, Penteriani V. 2013. Individual and spatio-temporal variations in the home range behaviour of a long-lived, territorial species. Oecologia. 172:371–385. 10.1007/s00442-012-2493-7 23086505

[CIT0022] Church KDW , Grant JWA. 2019a. Effects of habitat complexity, dominance and personality on habitat selection: ideal despotic cichlids. Ethology. 125:832–845. 10.1111/eth.12938

[CIT0023] Church KDW , Grant JWA. 2019b. Ideal despotic distributions in convict cichlids (*Amatitlania nigrofasciata*)? Effects of predation risk and personality on habitat preference. Behav Process. 158:163–171. 10.1016/j.beproc.2018.12.002 30529688

[CIT0024] Collinge SK. 2000. Effects of grassland fragmentation on insect species loss, colonization, and movement patterns. Ecology. 81:2211–2226. 10.2307/177109

[CIT0025] Cooper SJ. 2000. Seasonal energetics of mountain chickadees and juniper titmice. Condor. 102:635–644. 10.1650/0010-5422(2000)102[0635:seomca]2.0.co;2

[CIT0026] Cooper SJ , Sonsthagen S. 2007. Heat production from foraging activity contributes to thermoregulation in black-capped chickadees. Condor. 109:446–451. 10.1650/0010-5422(2007)109[446:hpffac]2.0.co;2

[CIT0027] Cumming G , Finch S. 2005. Inference by eye: confidence intervals and how to read pictures of data. Am Psychol. 60:170–180. 10.1037/0003-066X.60.2.170 15740449

[CIT0028] Desrochers A. 1989. Sex, dominance, and microhabitat use in wintering black-capped chickadees: a field experiment. Ecology. 70:636–645. 10.2307/1940215

[CIT0029] Desrochers A , Fortin MJ. 2000. Understanding avian responses to forest boundaries: a case study with chickadee winter flocks. Oikos. 91:376–384. 10.1034/j.1600-0706.2000.910218.x

[CIT0030] Desrochers A , Hannon SJ, Nordin KE. 1988. Winter survival and territory acquisition in a northern population of black-capped chickadees. Auk. 105:727–736. 10.1093/auk/105.4.727

[CIT0031] Diffendorfer JE , Gaines MS, Holt RD. 1995. Habitat fragmentation and movements of three small mammals (*Sigmodon*, *Microtus*, and *Peromyscus*). Ecology. 76:827–839. 10.2307/1939348

[CIT0032] Dingemanse NJ , Kazem AJN, Réale D, Wright J. 2010. Behavioural reaction norms: animal personality meets individual plasticity. Trends Ecol Evol. 25:81–89. 10.1016/j.tree.2009.07.013 19748700

[CIT0033] Ficken MS , Weise CM, Popp JW. 1990. Dominance rank and resource access in winter flocks of black-capped chickadees. Wilson Bull. 102:623–633. https://www.jstor.org/stable/4162935.

[CIT0034] Found R , St. Clair CC. 2016. Behavioural syndromes predict loss of migration in wild elk. Anim Behav. 115:35–46. 10.1016/j.anbehav.2016.02.007

[CIT0035] Fox RA , Ladage LD, Roth TC II, Pravosudov VV. 2009. Behavioural profile predicts dominance status in mountain chickadees, *Poecile gambeli*. Anim Behav. 77:1441–1448. 10.1016/j.anbehav.2009.02.022 20161203 PMC2712732

[CIT0036] Fretwell SD , Lucas HL. 1969. On territorial behavior and other factors influencing habitat distribution in birds. Acta Biotheor. 19:16–36. 10.1007/bf01601953

[CIT0037] Gelman A. 2008. Scaling regression inputs by dividing by two standard deviations. Stat Med. 27:2865–2873. 10.1002/sim.3107 17960576

[CIT0038] Gelman A , Su Y-S. 2014. arm: Data analysis using regression and multilevel/hierarchical models. https://CRAN.R-project.org/package=arm.

[CIT0039] Greenland S , Senn SJ, Rothman KJ, Carlin JB, Poole C, Goodman SN, Altman DG. 2016. Statistical tests, P values, confidence intervals, and power: a guide to misinterpretations. Eur J Epidemiol. 31:337–350. 10.1007/s10654-016-0149-3 27209009 PMC4877414

[CIT0040] Griffiths R , Double MC, Orr K, Dawson RJG. 1998. A DNA test to sex most birds. Mol Ecol. 7:1071–1075. 10.1046/j.1365-294x.1998.00389.x 9711866

[CIT0041] Grubb TC. 1978. Weather-dependent foraging rates of wintering woodland birds. Auk. 95:370–376. https://www.jstor.org/stable/4085455

[CIT0042] Haave-Audet E , Martin JGA, Wijmenga JJ, Mathot KJ. 2024. Information gathering is associated with increased survival: a field experiment in black-capped chickadees (*Poecile atricapillus*). Am Naturalist. 203:109–123. 10.1086/727509 38207133

[CIT0043] Hadfield JD , Wilson AJ, Garant D, Sheldon BC, Kruuk LEB. 2010. The misuse of BLUP in ecology and evolution. Am Naturalist. 175:116–125. 10.1086/648604 19922262

[CIT0044] Hartig F. 2022. DHARMa: Residual diagnostics for hierarchical (Multi-Level / Mixed) regression models. https://CRAN.R-project.org/package=DHARMa.

[CIT0045] Hartzler JE. 1970. Winter dominance relationship in black-capped chickadees. Wilson Bull. 82:427–434. https://www.jstor.org/stable/4160013.

[CIT0046] Hertel AG , Niemelä PT, Dingemanse NJ, Mueller T. 2020. A guide for studying among-individual behavioral variation from movement data in the wild. Mov Ecol. 8:1–18. 10.1186/s40462-020-00216-8 32612837 PMC7325061

[CIT0047] Hogstad O. 2015. Rank-related response in foraging site selection and vigilance behaviour of a small passerine to different winter weather conditions. Ornis Fenn. 92:53–62. 10.51812/of.133868

[CIT0048] Holyoak M , Casagrandi R, Nathan R, Revilla E, Spiegel O. 2008. Trends and missing parts in the study of movement ecology. Proc Natl Acad Sci USA. 105:19060–19065. 10.1073/pnas.0800483105 19060194 PMC2614715

[CIT0049] Horton KG , Morris SR, Van Doren BM, Covino KM. 2023. Six decades of North American bird banding records reveal plasticity in migration phenology. J Anim Ecol. 92:738–750. 10.1111/1365-2656.13887 36655993

[CIT0050] Humphries MM , Careau V. 2011. Heat for nothing or activity for free? Evidence and implications of activity-thermoregulatory heat substitution. Integr Comp Biol. 51:419–431. 10.1093/icb/icr059 21700569

[CIT0051] Janin A , Léna JP, Joly P. 2012. Habitat fragmentation affects movement behavior of migrating juvenile common toads. Behav Ecol Sociobiol. 66:1351–1356. 10.1007/s00265-012-1390-8

[CIT0052] Jansson C , Ekman J, Vonbromssen A. 1981. Winter mortality and food supply in tits Parus spp. Oikos. 37:313–322. 10.2307/3544122

[CIT0053] Kays R , Crofoot MC, Jetz W, Wikelski M. 2015. Terrestrial animal tracking as an eye on life and planet. Science. 348:aaa2478. 10.1126/science.aaa2478 26068858

[CIT0054] Kerth G , Melber M. 2009. Species-specific barrier effects of a motorway on the habitat use of two threatened forest-living bat species. Biol Conserv. 142:270–279. 10.1016/j.biocon.2008.10.022

[CIT0055] Kessel B. 1976. Winter activity patterns of black-capped chickadees in interior Alaska. Wilson Bull. 88:36–61.

[CIT0056] Krama T , Krams R, Popovs S, Trakimas G, Rantala MJ, Freeberg TM, Krams IA. 2023. Permanent ad-lib feeders decrease the survival of wintering great tits (*Parus major*). Birds. 4:225–235. 10.3390/birds4020019

[CIT0057] Lahti K , Orell M, Rytkönen S, Koivula K. 1998. Time and food dependence in Willow Tit winter survival. Ecology. 79:2904–2916. 10.2307/176525

[CIT0058] LaRocque M , Arteaga-Torres JD, Sridharan S, Wijmenga JJ, Haave-Audet E, Mathot KJ. 2023. An investigation of personality-related recapture bias in black-capped chickadees, *Poecile atricapillus*. Anim Behav. 196:103–112. 10.1016/j.anbehav.2022.12.007

[CIT0059] LaRocque M , Wijmenga JJ, Mathot KJ. 2024. Age, sex and temperature shape off-territory feeder use in black-capped chickadees. Behav Ecol. 1. 10.5061/dryad.47d7wm3pn PMC11491524 39434963

[CIT0060] Latimer CE , Cooper SJ, Karasov WH, Zuckerberg B. 2018. Does habitat fragmentation promote climate-resilient phenotypes? Oikos. 127:1069–1080. 10.1111/oik.05111

[CIT0061] Lemmon D , Withiam ML, Barkan CPL. 1997. Mate protection and winter pair-bonds in black-capped chickadees. Condor. 99:424–433. 10.2307/1369949

[CIT0062] Lenda M , Maciusik B, Skorka P. 2012. The evolutionary, ecological and behavioural consequences of the presence of floaters in bird populations. North-Western J Zool. 8:394–408.

[CIT0063] Lewden A , Petit M, Vézina F. 2012. Dominant black-capped chickadees pay no maintenance energy costs for their wintering status and are not better at enduring cold than subordinate individuals. J Comp Physiol B Biochem Syst Environ Physiol. 182:381–392. 10.1007/s00360-011-0625-8 22037961

[CIT0064] Lima SL , Dill LM. 1990. Behavioral decisions made under the risk of predation: a review and prospectus. Can J Zool. 68:619–640. 10.1139/z90-092

[CIT0065] Mady RP , Hochachka WM, Bonter DN. 2021. Consistency in supplemental food availability affects the space use of wintering birds. Behav Ecol. 32:580–589. 10.1093/beheco/arab002

[CIT0066] Marsman M , Wagenmakers EJ. 2017. Three insights from a bayesian interpretation of the one-sided P value. Educ Psychol Meas. 77:529–539. 10.1177/0013164416669201 29795927 PMC5965556

[CIT0067] Mathot KJ , Arteaga-Torres JD, Wijmenga JJ. 2022. Individual risk-taking behaviour in black-capped chickadees (*Poecile atricapillus*) does not predict annual survival. R Soc Open Sci. 9:220299. 10.1098/rsos.220299 35911194 PMC9326292

[CIT0068] Matthews SA , Wong MYL. 2015. Temperature-dependent resolution of conflict over rank within a size-based dominance hierarchy. Behav Ecol. 26:947–958. 10.1093/beheco/arv042

[CIT0069] Morales JM , Moorcroft PR, Matthiopoulos J, Frair JL, Kie JG, Powell RA, Merrill EH, Haydon DT. 2010. Building the bridge between animal movement and population dynamics. Philos Trans R Soc London Ser B. 365:2289–2301. 10.1098/rstb.2010.0082 20566505 PMC2894961

[CIT0070] Nakagawa S , Schielzeth H. 2010. Repeatability for Gaussian and non‐Gaussian data: a practical guide for biologists. Biol Rev. 85:935–956. 10.1111/j.1469-185x.2010.00141.x 20569253

[CIT0071] Nathan R , Getz WM, Revilla E, Holyoak M, Kadmon R, Saltz D, Smouse PE. 2008. A movement ecology paradigm for unifying organismal movement research. Proc Natl Acad Sci USA. 105:19052–19059. 10.1073/pnas.0800375105 19060196 PMC2614714

[CIT0072] Neumann LK , Fuhlendorf SD, Davis CD, Wilder SM. 2022. Climate alters the movement ecology of a non-migratory bird. Ecol Evol. 12:e8869. 10.1002/ece3.8869 35475174 PMC9034450

[CIT0073] Newton I , Rothery P. 2001. Estimation and limitation of numbers of floaters in a Eurasian Sparrowhawk population. IBIS. 143:442–449. 10.1111/j.1474-919x.2001.tb04945.x

[CIT0074] Odum EP. 1942. Annual cycle of the black-capped chickadee: 3. Auk. 59:499–531. 10.2307/4079461

[CIT0075] Penteriani V , Ferrer M, Delgado MM. 2011. Floater strategies and dynamics in birds, and their importance in conservation biology: towards an understanding of nonbreeders in avian populations. Anim Conserv. 14:233–241. 10.1111/j.1469-1795.2010.00433.x

[CIT0076] Poessel SA , Burdett CL, Boydston EE, Lyren LM, Alonso RS, Fisher RN, Crooks KR. 2014. Roads influence movement and home ranges of a fragmentation-sensitive carnivore, the bobcat, in an urban landscape. Biol Conserv. 180:224–232. 10.1016/j.biocon.2014.10.010

[CIT0077] Purchase CF , Hutchings JA. 2008. A temporally stable spatial pattern in the spawner density of a freshwater fish: evidence for an ideal despotic distribution. Can J Fish Aquat Sci. 65:382–388. 10.1139/f07-171

[CIT0078] R Development Core Team. 2020. R: a language and environment for statistical computing. 4.0.3 ed. Vienna, Austria: R Foundation for Statistical Computing. http://www.R-project.org.

[CIT0079] R Studio Team. 2020. RStudio: integrated development for R. Boston (MA): RStudio, PBC.

[CIT0080] Ramsay SM , Ratcliffe LM. 2003. Determinants of social rank in female black-capped chickadees (*Poecile atricapilla*). Can J Zool. 81:117–121. 10.1139/z02-241

[CIT0081] Robles H , Ciudad C. 2017. Floaters may buffer the extinction risk of small populations: an empirical assessment. Proc R Soc B. 284:20170074. 10.1098/rspb.2017.0074 PMC5413921 28424345

[CIT0082] Rohwer VG , Rohwer S, Wingfield JC. 2020. Despotic aggression in pre-moulting painted buntings. R Soc Open Sci. 7:191510. 10.1098/rsos.191510 32257318 PMC7062092

[CIT0083] Rus AI , McArthur C, Mella VSA, Crowther MS. 2021. Habitat fragmentation affects movement and space use of a specialist folivore, the koala. Anim Conserv. 24:26–37. 10.1111/acv.12596

[CIT0085] Sells SN , Mitchell MS, Ausband DE, Luis AD, Emlen DJ, Podruzny KM, Gude JA. 2022. Economical defence of resources structures territorial space use in a cooperative carnivore. Proc R Soc B. 289:20212512. 10.1098/rspb.2021.2512 PMC8753142 35016539

[CIT0086] Smith DC , Van Buskirk J. 1988. Winter territoriality and flock cohesion in the black-capped chickadee *Parus atricapillus*. Anim Behav. 36:466–476. 10.1016/s0003-3472(88)80017-4

[CIT0087] Smith SM. 1976. Ecological aspects of dominance hierarchies in black-capped chickadees. Auk. 93:95–107. https://www.jstor.org/stable/4084835.

[CIT0088] Smith SM. 1984. Flock switching in chickadees: why be a winter floater? Am Nat. 123:81–98. 10.1086/284188

[CIT0089] Smith SM. 1992. The black-capped chickadee: behavioral ecology and natural history. Ithaca (NY): Cornell 466 University Press.

[CIT0090] Smith SM. 1994. Social influences on the dynamics of a northeastern black‐capped chickadee population. Ecology. 75:2043–2051. 10.2307/1941609

[CIT0091] Smith SM. 1997. Black-capped chickadee. Mechanicsburg (PA): Stackpole Books.

[CIT0092] Spiegel O , Leu ST, Bull CM, Sih A. 2017. What’s your move? Movement as a link between personality and spatial dynamics in animal populations. Ecology Lett. 20:3–18. 10.1111/ele.12708 28000433

[CIT0093] Sridharan S. 2021. Investigating the value of incorporating behavioural measures in a discriminant function developed for sex assignment in black-capped chickadees (*Poecile atricapillus*) [thesis]. [Edmonton]: University of Alberta.

[CIT0094] Stoffel MA , Nakagawa S, Schielzeth H. 2017. rptR: repeatability estimation and variance decomposition by generalized linear mixed-effects models. Methods Ecol Evol. 8:1639–1644. 10.1111/2041-210x.12797

[CIT0095] Stuber EF , Carlson B, Jesmer BR. 2022. Spatial personalities: a meta-analysis of consistent individual differences in spatial behavior. Behav Ecol. 33:477–486. 10.1093/beheco/arab147

[CIT0096] Studd EK , Bates AE, Bramburger AJ, Fernandes T, Hayden B, Henry HAL, Humphries MM, Martin R, McMeans BC, Moise ERD, et al. 2021. Nine maxims for the ecology of cold-climate winters. BioScience. 71:820–830. 10.1093/biosci/biab032

[CIT0097] Sutton AO , Studd EK, Fernandes T, Bates AE, Bramburger AJ, Cooke SJ, Hayden B, Henry HAL, Humphries MM, Martin R, et al. 2021. Frozen out: unanswered questions about winter biology. Environ Rev. 29:431–442. 10.1139/er-2020-0127

[CIT0098] Turcotte Y , Desrochers A. 2005. Landscape-dependent distribution of northern forest birds in winter. Ecography. 28:129–140. 10.1111/j.0906-7590.2005.04047.x

[CIT0099] Weise CM , Meyer JR. 1979. Juvenile dispersal and development of site-fidelity in the black-capped chickadee. Auk. 96:40–55. 10.1093/auk/96.1.40

[CIT0100] Wickham H. 2016. ggplot2: Elegant graphics for data analysis. 2nd ed. New York: Springer.

[CIT0101] Wilson WH. 2001. The effects of supplemental feeding on wintering black-capped chickadees (*Poecile atricapilla*) in central Maine: population and individual responses. Wilson Bull. 113:65–72. 10.1676/00435643(2001)113[0065:TEOSFO]2.0.CO;2

