# Supplementary material for: Age, sex, and temperature shape off-territory feeder use in black-capped chickadees
Source: Behav Ecol. 2024 Oct 3;35(6):arae080. doi: 10.1093/beheco/arae080 (PMC11491524; doi:10.1093/beheco/arae080)
Supplement: arae080_suppl_Supplementary_Material [file arae080_suppl_supplementary_material.docx]

**Supplementary Materials for:** Age, sex and temperature shape off-territory feeder use in black-capped chickadees

**Authors:** Megan LaRocque^1,*^, Jan J. Wijmenga^1^, and Kimberley J. Mathot^1,2^

^1^ Department of Biological Sciences, University of Alberta, Edmonton, AB T6G 2E9. Canada

^2^ Canada Research Chair in Integrative Ecology

*Author for correspondence: mgfroese@ualberta.ca

Contents

[Supplementary Text S1. Discriminant function for chickadee sex assignment. 5](#_Toc176941437)

[Supplementary Text S2. Exclusion of thermal feeder. 5](#_Toc176941438)

[Supplementary Text S3. Analyses with versus without data from February 15^th^ through 28^th^, 2023. 5](#_Toc176941439)

[Supplementary Text S4. Investigating model fit. 6](#_Toc176941440)

[Supplementary Text S5. Rarefaction analysis. 6](#_Toc176941441)

[Supplementary Text S6. Age-Sex differences in annual survival. 7](#_Toc176941442)

[Table S1. Model results for probability of foraging off-territory (binomial GLMM) and daily feeder visits (LMM) from January 9^th^ to February 14^th^, 2023 (inclusive), and February 24^rd^ to 28th, 2023 (inclusive). Note, dates from February 15^th^ to 23^rd^, 2023, are not included because one feeder had a broken circuit board during this time. Proportion overlap (pr) are reported for estimates with CrIs that overlap zero and report the proportion of estimate values which overlap zero. See Tables S2 and S3 below for pr values of pairwise contrasts. 8](#_Toc176941443)

[Table S2. Proportion overlap (pr) values for pairwise contrasts for each Age-Sex category for the off-territory model from January 9^th^ to February 14^th^, 2023 (inclusive), and February 24^rd^ to 28th, 2023 (inclusive). Note, dates from February 15^th^ to 23^rd^, 2023, are not included because one feeder had a broken circuit board during this time (see Table S2 above for model output). Estimated differences are calculated by subtracting row headings from column headings. Above the diagonal are intercept contrasts and below the diagonal are slope contrasts (i.e., temperature interaction). 9](#_Toc176941444)

[Table S3. Proportion overlap (pr) values for pairwise contrasts for each Age-Sex category for the square root of daily feeder visits model from January 9^th^ to February 14^th^, 2023 (inclusive), and February 24^rd^ to 28th, 2023 (inclusive). Note, dates from February 15^th^ to 23^rd^, 2023, are not included because one feeder had a broken circuit board during this time (see Table S2 above for model output). Estimated differences are calculated by subtracting row headings from column headings. Above the diagonal are intercept contrasts and below the diagonal are slope contrasts (i.e., temperature interaction). 10](#_Toc176941445)

[Table S4. Comparison of daily feeder visit model outputs. *Note that this repeatability estimate is not adjusted repeatability. See Table S5 for pairwise comparison of CrIs. See Figures S2-S7 for associated DHARMa outputs. Note that the model including IxE produces negative estimates for variance components (Bird ID), which is an indication that the model is poorly specified to the data. 11](#_Toc176941446)

[Table S5. Proportion overlap (pr) values for pairwise contrasts for daily feeder visit models. Estimated differences are calculated by subtracting the two estimates in the “Contrasts” column as shown. The first four rows are for intercept contrasts and the last four rows are for slope (i.e., temperature interaction) contrasts. Bolded contrasts are those that have different p-value significances across models. However, it is important to note that these differences do not change our interpretation of our results. The effects contrasts show that in some models, the strength of the negative relationship between temperature and daily feeder visits differs between Age-Sex categories; however, as discussed in the main text, each Age-Sex category still has a significant negative relationship. See Table S4 for model output. FJ = female, juvenile; FA = female, adult; MJ = male, juvenile; MA = male, adult; T = temperature interaction. 13](#_Toc176941447)

[Table S6. Off-territory model comparison with and without random slopes (IxE). See Table S5 for pairwise comparison proportion overlap (pr) values. See Figure S10 for DHARMa output. 15](#_Toc176941448)

[Table S7. Effect size contrasts for off-territory models with and without random slopes (IxE). Bolded pr-values are those that have a different pr-value strength of support in the IxE model. However, it is important to note that these differences do not change our interpretation of our results. The effects contrasts show that in the IxE model, the strength of the negative relationship between temperature and off-territory use differs between juvenile females and males; however, as discussed in the main text, each Age-Sex category still has a significant negative relationship. See Table S6 for model outputs. FJ = female, juvenile; FA = female, adult; MJ = male, juvenile; MA = male, adult; T = temperature interaction. 17](#_Toc176941449)

[Table S8. Rarefication results for GLMM models of off-territory use (1 = off territory feeders used, 0 = no off-territory feeders used) after removal of a single feeder (and all birds for which that feeder was the core feeder). Models were constructed with binomial error distribution. Proportion overlap (pr) values are provided for effect sizes with CrIs that overlap zero to show the proportion of estimates which overlap zero. See Table S9 below for proportion overlap (pr) of pairwise contrasts. 19](#_Toc176941450)

[Table S9. Proportion overlap (pr) values for pairwise contrasts of estimates for off-territory use rarefaction analyses presented in Table S8. Off-territory use (1 = off territory feeders used, 0 = no off-territory feeders used) GLMM models were constructed with binomial error distribution. Estimated differences are calculated by subtracting the two estimates in the “Contrasts” column as shown. FJ = female, juvenile; FA = female, adult; MJ = male, juvenile; MA = male, adult; T = temperature interaction. 21](#_Toc176941451)

[Table S10. Rarefication results for LMM models of square root (daily feeder visits) after removal of a single feeder (and all birds for which that feeder was the core feeder). See Table S11 below for proportion overlap (pr) values of pairwise contrasts. 23](#_Toc176941452)

[Table S11. Proportion overlap (pr) values for pairwise contrasts of estimates for feeder visit rate rarefaction analyses presented in Table S10. Estimated differences are calculated by subtracting the two estimates in the “Contrasts” column as shown. FJ = female, juvenile; FA = female, adult; MJ = male, juvenile; MA = male, adult; T = temperature interaction. 25](#_Toc176941453)

[Figure S1. Distribution of total visit counts summed by day (grey bars) and average daily temperature in °C (black dots) across the study period. There was a circuit board failure between February 18^th^ and 23^rd^, 2023, resulting in missing data for this period. 27](#_Toc176941454)

[Figure S2. DHARMa results for the square-root LMM for daily feeder visits (presented in main text). Note that visual inspection of the plots shows: 1) left plot – minimal deviation in the QQ plot away from the red 1:1 line, 2) center plot – no significant deviation in the dispersion of residuals as indicated by the red vertical line falling near the mean of the residuals, and 3) right plot - evidence of outliers in residuals. Importantly, outlier residuals are always under-represented. Underrepresentation of outliers is generally not problematic as it reduces power and leads to conservative conclusions. 28](#_Toc176941455)

[Figure S3. DHARMa results for a non-transformed LMM for daily feeder visits. Note that visual inspection of the plots shows: 1) left plot - deviation in the QQ plot away from the red 1:1 line, 2) center plot - significant deviation in the dispersion of residuals as indicated by the red vertical line not falling near the mean of the residuals, and 3) right plot - evidence of outliers in residuals. Importantly, small residuals are over-represented, which can lead to underestimation of errors and therefore, overconfidence in estimate effects. Larger residuals are under-represented, which is generally not problematic as it reduces power and leads to conservative conclusions. 29](#_Toc176941456)

[Figure S4. DHARMa results for including random slopes (IxE) in the square root transformed LMM for daily feeder visits. Note that visual inspection of the plots shows: 1) left plot - deviation in the QQ plot away from the red 1:1 line, 2) center plot – no significant deviation in the dispersion of residuals as indicated by the red vertical line falling near the mean of the residuals, and 3) right plot - no evidence of outliers in residuals. 30](#_Toc176941457)

[Figure S5. DHARMa results for a quadratic LMM for daily feeder visits. Note that visual inspection of the plots shows: 1) left plot - deviation in the QQ plot away from the red 1:1 line, 2) center plot – no significant deviation in the dispersion of residuals as indicated by the red vertical line falling near the mean of the residuals, and 3) right plot - evidence of outliers in residuals. Importantly, large residuals are over-represented (indicated by red bar on far right of histogram), which can lead to underestimation of errors and therefore, overconfidence in estimate effects. 31](#_Toc176941458)

[Figure S6. DHARMa results for a negative binomial GLMM for daily feeder visits. Note that visual inspection of the plots shows: 1) left plot – small deviation in the QQ plot away from the red 1:1 line, 2) center plot - significant deviation in the dispersion of residuals as indicated by the red vertical line not falling near the mean of the residuals, and 3) right plot - evidence of outliers in residuals. Importantly, small residuals outliers are over-represented, which can lead to underestimation of errors and therefore, overconfidence in estimate effects. Larger residuals outliers are under-represented, which is generally not problematic as it reduces power and leads to conservative conclusons. 32](#_Toc176941459)

[Figure S7. DHARMa results for the off-territory model (a) excluding (as presented in the main text) and (b) including IxE. Note that visual inspection of the plots shows: 1) left plot - deviation in the QQ plot away from the red 1:1 line for both models, but more marked deviation whenincluding IxE (lower panel), 2) center plot – no significant deviation in the dispersion of residuals as indicated by the red vertical line falling near the mean of the residuals, and 3) right plot – no evidence of outliers in residuals. 33](#_Toc176941460)

[Supplementary References 34](#_Toc176941461)

# **Supplementary Text S1. Discriminant function for chickadee sex assignment**.

For individuals in our dataset without molecular sex data (N=13), we used a discriminant function developed by Sridharan (2021) to assign a sex, when possible. This discriminant function was developed specifically for our population of black-capped chickadees based on a sample of 469 birds (238 females and 231 males). The discriminant function incorporates highly repeatable morphological traits (i.e., body mass, wing length, and tarsus length) as outlined in the following equation:

LD2 = 0.55886 (Body mass) + 1.0064 (Wing length) + 0.28042 (Tarsus length).

For those individuals who we had repeated morphological measures, we calculated the average of each trait measurement. Birds can conclusively be assigned ‘male’ if the discriminant function score is >81 and ‘female’ if the score is <77. Individuals who have discriminant scores (DS) that fall in the intermediate range (77 ≤ DS ≤ 81) cannot be conclusively assigned as male or female. However, scores at the lower end of this range are more likely to be female (only 4% of individuals with scores of 77-78 were male) while scores at the higher end are more likely to be male (68% of individuals with scores of 80-81 were male). When we used the discriminant function, we removed individuals who did not have conclusive sex assignment (N=3). Therefore, the remaining individuals (N=10) could be assigned either ‘male’ or ‘female’ with 100% accuracy.

# Supplementary Text S2. Exclusion of thermal feeder.

In the study year, 1 of 8 regular feeder locations within the study area was equipped with a different frequency RFID system as part of another study aimed at understanding variation in body temperature in chickadees (Feeder 14 in Figure 1 of main text, referred to as “thermal feeder”). This system was installed from December 4^th^, 2022, to March 17^th^, 2023, and required a different type of PIT tag and RFID antenna frequency, which meant that the majority of birds in the marked population could not be detected at the thermal feeder during the relevant dates for the present study. We relied on the feeder visits recorded between October 29^th^ and December 4^th^, 2022 (inclusive), across all feeders to filter out birds that were known to have used the thermal feeder location from our study sample (N=32). Furthermore, we removed N=1 individual that was initially identified (i.e., tagged and given a unique ID) at this feeder after December 4^th^. Although we cannot exclude the possibility that remaining birds in our dataset commenced using the thermal feeder after December 4^th^, if they did, this would mean that our estimates of within- and among-individual propensity to forage off-territory are underestimated, and thus make our results conservative.

# Supplementary Text S3. Analyses with versus without data from February 15^th^ through 28^th^, 2023.

Analyses presented in the main text include data from January 9 to February 14, 2023 (inclusive). We excluded February 15^th^ to February 28^th^, 2023 (inclusive) because there was a gap in data from February 15^th^ to February 23^rd^, 2023 (inclusive) as one of the 8 feeders had a damaged circuit board during this period. Furthermore, chickadees can begin to establish breeding territories in early February, which can initiate winter flock break up (Smith, 1992). Thus, for the analysis presented in the main test, we did not include February 24^th^ to 28^th^, 2023 (inclusive).

To confirm that this did not unduly influence results, we ran analyses that did include the dates from February 15^th^ to 28^th^, 2023 (inclusive). We found evidence that indeed an alternative biological process is occurring during February 24^th^ to 28^th^, 2023 (inclusive). The temperature effects were diminished (see Supplementary Tables S1-S3 for model output and pairwise contrasts), however, we found that on the coldest day in our dataset (-26.8°C on February 24^th^), there was low feeder visitation (see Supplementary Figure S1). This suggests that during this time, chickadees are less dependent on the feeders regardless of temperature (i.e., flock break up has commenced).

# Supplementary Text S4. Investigating model fit.

To ensure that our models for daily feeder visits and off-territory use achieved good model fit and that model structure did not unduly influence our interpretations, we constructed multiple alternative models and compared model output and model fit using the ‘DHARMa’ package (v. 0.4.6; Hartig 2022). All alternative models included the same fixed and random effects as presented in the main text Methods section, but included addition fixed and/or random effects, or were modelled with different error families. Models presented in the main text were the models that achieved the best fit based on visual inspection of plots using DHARMa.

For daily feeder visits, we considered the following model structures: 1) LMER model with square root transformed count of feeder visits, with a gaussian error distribution, 2) LMER model with non-transformed count of feeder visits, with a gaussian error distribution, 3) LMER model with non-transformed count of feeder visits, with the additional random slopes for temperature (IxE), with a gaussian error distribution, 4) LMER model with non-transformed count of feeder visits, with an additional fixed effect ‘temperature^2^’, with a gaussian error distribution, and 5) GLMM of count of feeder visits, with a negative binomial error family. All models yielded qualitatively similar interpretations of the factors that we were a priori interested in studying (temperature and age-sex) (see Tables S4 and S5). The LMER model with square root transformed count of feeder visits achieved the best model fit (see Figures S2-S6) and is presented in the main text.

Off-territory feeder (1= yes, 0 = no), use was modelled with binomial errors and exhibit good model fit. However, we additionally considered a random slope (IxE) model. Both models resulted in comparable biological interpretations (see Table S6 for model output and Table S7 for pairwise estimate comparisons), however, model fit was markedly better for the model that did not include IxE (see Table S6 for negative variance components of off-territory use and Figure S7 for DHARMa output) and is presented in the main text.

# Supplementary Text S5. Rarefaction analysis.

We conducted rarefaction analyses to address the possibility specific feeders were responsible for the overall patterns reported in the main text. We excluded each feeder location one-by-one and re-ran the analyses described above, resulting in a total of 7 rarefactions. For each rarefaction, all birds for which the excluded feeder was their core feeder were removed from the dataset. The two birds in the dataset which had two feeders defined as being their core feeders, were not removed in any rarefaction, as their one of their core feeders was always included with the removal of a single feeder. We found that regardless of the feeder that was excluded, feeder visit rate results were quantitively similar (see Tables S8-S11 for model results and pairwise comparisons). Some differences existed in the significance of pairwise contrasts; however, the contrasts in question where different depending on which feeder location was removed. Therefore, these qualitative differences were not generalizable. We found that the patterns for probability of off-territory feeder use remained qualitatively similar with respect to our covariates (age and sex) across most rarefactions, but some qualitative differences were observed for specific Age-Sex categories when we removed F04, F09, and F11. However, the age-sex category in question differed for different rarefactions suggesting that this was not a generalizable difference. Some differences also existed in the significance of pairwise contrasts; however, the contrasts in question where different depending on which feeder location was removed. Therefore, these qualitative differences were not generalizable. Overall, we found that ‘core feeder’ location did not intrinsically influence the patterns that we see.

# Supplementary Text S6. Age-Sex differences in annual survival.

Given that we observed age- and sex-related differences in off-territory use and daily feeder visits and found that greater off-territory feeder use predicts a lower survival probability, we wanted to assess differences across our Age-Sex groups. Our sample sizes for each Age-Sex group precluded us from performing rigorous analyses, however we present the Age-Sex survival rates below.

Juvenile females had a survival rate of 59.46% (N_survived_=22, N_died_=15), juvenile males had a survival rate of 51.52% (N_survived_=17, N_died_=16), adult females had a survival rate of 65.63% (N_survived_=21, N_died_=11), and adult males had a survival rate of 55.56% (N_survived_=20, N_died_=16)

# Table S1. Model results for probability of foraging off-territory (binomial GLMM) and daily feeder visits (LMM) from January 9^th^ to February 14^th^, 2023 (inclusive), and February 24^rd^ to 28th, 2023 (inclusive). Note, dates from February 15^th^ to 23^rd^, 2023, are not included because one feeder had a broken circuit board during this time. Proportion overlap (pr) are reported for estimates with CrIs that overlap zero and report the proportion of estimate values which overlap zero. See Tables S2 and S3 below for pr values of pairwise contrasts.

|  | Probability of foraging off territory | Daily feeder visits |  |
| --- | --- | --- | --- |
| *Fixed effects* | *β (95% CrI)* | *β (95% CrI)* |  |
| Female – Juvenile^1^ | -2.52 (-4.53, -0.28) | 8.13 (7.68, 8.82) |  |
| Female – Adult^1^ | -3.10 (-4.39, -0.82) | 7.77 (7.04, 8.22) |  |
| Male – Juvenile^1^ | -3.10 (-4.50, -0.58) | 9.49 (8.92, 10.15) |  |
| Male – Adult^1^ | -4.66 (-5.42, -1.53) | 9.03 (8.51, 9.65) |  |
| Female – Juvenile : Temperature | -0.22 (-0.59, 0.19)  *pr = 0.18* | -0.41 (-0.58, -0.21) |  |
| Female – Adult : Temperature | -0.15 (-0.63, 0.17)  *pr = 0.18* | -0.01 (-0.22, 0.14)  *pr = 0.38* |  |
| Male – Juvenile : Temperature | 0.18 (0.06, 0.82) | -0.86 (-0.99, -0.63) |  |
| Male – Adult : Temperature | 0.54 (-0.22, 0.68)  *pr = 0.11* | -0.54 (-0.73, -0.36) |  |
| *Random Effects* | σ *(95% CrI)* | σ *(95% CrI)* |  |
| Bird ID N = 138 | 4.05 (3.28, 4.92) | 1.96 (1.77, 2.22) |  |
| Core Feeder N = 8 | 11.62 (2.88, 20.61) | 0.05 (0.02, 0.09) | |
| Residual N= 5597 | 1 (--)^2^ | 2.48 (2.41, 2.59) |  |
| *Repeatability* | *r (95% CrI)* | *r (95% CrI)* |  |
| Transponder Hex Code N=138 | 0.55 (0.51, 0.60) | 0.43 (0.36, 0.49) |  |

^1^Note, intercept values for Age-Sex categories are estimated at -26.8°C (the lowest temperature in our data set containing all dates), and temperature was standardized prior to analysis, therefore estimate effect sizes are for 1 s.d. change in temperature (i.e., 5.74°C).

^2^Residual variance is fixed to 1 for binary traits.

# Table S2. Proportion overlap (pr) values for pairwise contrasts for each Age-Sex category for the off-territory model from January 9^th^ to February 14^th^, 2023 (inclusive), and February 24^rd^ to 28th, 2023 (inclusive). Note, dates from February 15^th^ to 23^rd^, 2023, are not included because one feeder had a broken circuit board during this time (see Table S2 above for model output). Estimated differences are calculated by subtracting row headings from column headings. Above the diagonal are intercept contrasts and below the diagonal are slope contrasts (i.e., temperature interaction).

|  | Female-Juvenile | Female-Adult | Male-Juvenile | Male-Adult |
| --- | --- | --- | --- | --- |
| Female-Juvenile | --------------------------- | -0.22 (-2.00, 1.07)  *pr=0.38* | -1.53 (-3.16, -0.27)  *pr=0.02* | -0.04 (-1.57, 1.38)  *pr=0.48* |
| Female-Adult | 0.02 (-0.50, 0.49)  *pr=0.47* | ----------------------------- | -0.02 (-1.89, 1.03)  *pr=0.38* | -1.52 (-2.76, -0.01)  *pr=0.02* |
| Male-Juvenile | -0.26 (-0.98, 0.18)  *pr=0.10* | -0.74 (-1.11, -0.04)  *pr=0.01* | --------------------------- | -1.34 (-3.06, 0.27)  *pr=0.07* |
| Male-Adult | -0.51 (-1.06, 0.14)  *pr=0.06* | -0.84 (-1.06, 0.12)  *pr=0.05* | 0.01 (-0.45, 0.83)  *pr=0.34* | ------------------------ |

# Table S3. Proportion overlap (pr) values for pairwise contrasts for each Age-Sex category for the square root of daily feeder visits model from January 9^th^ to February 14^th^, 2023 (inclusive), and February 24^rd^ to 28th, 2023 (inclusive). Note, dates from February 15^th^ to 23^rd^, 2023, are not included because one feeder had a broken circuit board during this time (see Table S2 above for model output). Estimated differences are calculated by subtracting row headings from column headings. Above the diagonal are intercept contrasts and below the diagonal are slope contrasts (i.e., temperature interaction).

|  | Female-Juvenile | Female-Adult | Male-Juvenile | Male-Adult |
| --- | --- | --- | --- | --- |
| Female-Juvenile | ---------------------------- | -0.49 (-1.38, 0.14)  *pr=0.06* | 0.94 (0.01, 1.51)  *pr=0.02* | 1.94 (1.09, 2.66)  *pr=0.00* |
| Female-Adult | -0.39 (-1.22, 0.31)  *pr=0.01* | ----------------------------- | 1.37 (0.51, 2.02)  *pr=0.00* | 1.38 (0.70, 2.15)  *pr=0.00* |
| Male-Juvenile | 0.15 (-0.14, 0.40)  *pr=0.16* | 0.76 (0.52, 1.02)  *pr=0.00* | --------------------------- | -0.38 (-1.22, 0.31)  *pr=0.14* |
| Male-Adult | 0.48 (0.26, 0.77)  *pr=0.00* | 0.49 (0.26, 0.77)  *pr=0.00* | -0.29 (-0.51, 0.01)  *pr=0.02* | ------------------------ |

# Table S4. Comparison of daily feeder visit model outputs. *Note that this repeatability estimate is not adjusted repeatability. See Table S5 for pairwise comparison of CrIs. See Figures S2-S7 for associated DHARMa outputs. Note that the model including IxE produces negative estimates for variance components (Bird ID), which is an indication that the model is poorly specified to the data.

|  | Square root LMM (as presented in main text) | Non-transformed LMM | LMM + IxE | Quadratic LMM | Negative Binomial |
| --- | --- | --- | --- | --- | --- |
| *Fixed effects* | *β (95% CrI)* | *β (95% CrI)* | *β (95% CrI)* | *β (95% CrI)* | *β (95% CrI)* |
| Female – Juvenile | 8.67  (8.13, 8.95) | 73.45  (66.75, 86.06) | 72.57  (67.39, 82.58) | 84.81  (74.06, 93.06) | 4.32  (4.19, 4.46) |
| Female – Adult | 8.32  (7.77, 8.71) | 73.24  (62.08, 81.37) | 72.97  (61.98, 79.51) | 77.40  (69.78, 88.99) | 4.24  (4.13, 4.40) |
| Male – Juvenile | 9.63  (9.08, 9.98) | 91.49  (85.26, 105.11) | 91.21  (85.04, 101.45) | 101.73  (92.46, 112.80) | 4.53  (4.43, 4.70) |
| Male – Adult | 9.43  (8.95, 9.82) | 88.71  (82.00, 100.45) | 90.26  (82.07, 97.49) | 97.00  (86.84, 105.23) | 4.50  (4.38, 4.65) |
| Female – Juvenile : Temperature | -1.05  (-1.50, -0.65) | -15.38  (-18.23, -12.25) | -15.44  (-22.12, -8.02) | -18.54  (-20.97, -15.22) | -0.29  (-0.34, -0.25) |
| Female – Adult : Temperature | -0.79  (-1.25, -0.31) | -10.52  (-12.83, -6.45) | -9.53  (-17.27, -1.39) | -12.23  (-15.63, -9.15) | -0.24  (-0.28, -0.19) |
| Male – Juvenile : Temperature | -1.70  (-2.06, -1.10) | -22.42  (-25.57, -19.24) | -24.62  (-31.54, -16.14) | -24.88  (-28.53, -22.09) | -0.38  (-0.43, -0.33) |
| Male – Adult : Temperature | -1.24  (-1.77, -0.86) | -18.52  (-21.15, -14.93) | -18.56  (-25.46, -10.38) | -20.82  (-23.55, -17.44) | -0.34  (-0.38, -0.28) |
| *Random Effects* | *σ (95% CrI)* | *σ (95% CrI)* | *σ (95% CrI)* | *σ (95% CrI)* | *σ (95% CrI)* |
| Bird ID N=138 | 2.06  (1.85, 2.34) | 568.69  (518.63, 651.33) | -66.42  (-118.20, 552.55) | 586.53  (525.06, 650.14) | 0.13  (0.12, 0.14) |
| Core Feeder ID N=7 | 0.07  (0.03, 0.11) | 21.34  (11.50, 35.25) | 1.67  (0.50, 5.89) | 19.92  (11.49, 36.84) | 0.003  (0.001, 0.01) |
| Residual N=4969 | 1.87  (1.80, 1.95) | 511.19  (489.94, 529.46) | 439.41  (428.65, 460.97) | 482.39  (464.34, 500.53) | 1 (--) |
| *Repeatability* | *r (95% CrI)* | *r (95% CrI)* | *r (95% CrI)* | *r (95% CrI)* | *r (95% CrI)* |
| Bird ID N=138 | 0.50  (0.43, 0.57) | 0.51  (0.43, 0.58) | 0.48*  (-0.31, 0.57) | 0.52  (0.44, 0.59) | No appropriate means of calculating |

# Table S5. Proportion overlap (pr) values for pairwise contrasts for daily feeder visit models. Estimated differences are calculated by subtracting the two estimates in the “Contrasts” column as shown. The first four rows are for intercept contrasts and the last four rows are for slope (i.e., temperature interaction) contrasts. Bolded contrasts are those that have different p-value significances across models. However, it is important to note that these differences do not change our interpretation of our results. The effects contrasts show that in some models, the strength of the negative relationship between temperature and daily feeder visits differs between Age-Sex categories; however, as discussed in the main text, each Age-Sex category still has a significant negative relationship. See Table S4 for model output. FJ = female, juvenile; FA = female, adult; MJ = male, juvenile; MA = male, adult; T = temperature interaction.

|  | Square Root LMM (as presented in main text) | Non-transformed LMM | LMM + IxE | Quadratic LMM | Negative Binomial |
| --- | --- | --- | --- | --- | --- |
| *Contrast* | *β (95% CrI)*  *Proportion overlap* | *β (95% CrI)*  *Proportion overlap* | *β (95% CrI)*  *Proportion overlap* | *β (95% CrI)*  *Proportion overlap* | *β (95% CrI)*  *Proportion overlap* |
| FA – FJ | -0.41 (-0.93, 0.47)  *pr=0.24* | -4.93 (-15.12, 8.23)  *pr=0.24* | -4.35 (-15.64, 5.53)  *pr=0.20* | -4.03 (-15.17, 8.18)  *pr=0.28* | -0.04 (-0.23, 0.12)  *pr=0.23* |
| MJ – FJ | 0.71 (0.07, 1.47)  *pr=0.01* | 13.75 (3.01, 25.30)  *pr=0.00* | 16.08 (3.54, 25.63)  *pr=0.00* | 13.81 (2.10, 24.76)  *pr=0.01* | 0.20 (0.00, 0.34)  *pr=0.02* |
| MJ – FA | 1.12 (0.23, 1.65)  *pr=0.00* | 17.52 (6.65, 29.61)  *pr=0.00* | 17.44 (7.25, 28.89)  *pr=0.00* | 18.51 (6.93, 29.51)  *pr=0.00* | 0.23 (0.05, 0.40)  *pr=0.01* |
| MA – FJ | 1.07 (0.54, 1.95)  *pr=0.00* | 19.63 (10.31, 34.35)  *pr=0.00* | 19.88 (10.31, 34.34)  *pr=0.00* | 23.47 (9.41, 33.59)  *pr=0.00* | 0.30 (0.10, 0.46)  *pr=0.00* |
| MA – FA | 0.98 (0.24, 1.74)  *pr=0.00* | 17.85 (5.54, 29.62)  *pr=0.00* | 16.28 (6.48, 29.82)  *pr=0.00* | 16.69 (3.70, 27.34)  *pr=0.00* | 0.27 (0.08, 0.43)  *pr=0.00* |
| MA – MJ | -0.37 (-0.79, 0.58)  *pr=0.30* | -4.00 (-15.07, 9.28)  *pr=0.26* | -5.44 (-14.48, 7.34)  *pr=0.28* | -7.61 (-15.67, 8.09)  *pr=0.21* | -0.07 (-0.22, 0.13)  *pr=0.30* |
| **FJ:T – FA:T** | -0.23 (-0.55, -0.02)  *pr=0.02* | -5.30 (-10.26, -1.44)  *pr=0.00* | -6.42 (-16.14, 5.24)  *pr=0.16* | -6.37 (-9.80, -1.06)  *pr=0.00* | -0.06 (-0.12, 0.01)  *pr=0.06* |
| **FJ:T – MJ:T** | 0.20 (-0.05, 0.45)  *pr=0.06* | 3.53 (-1.35, 7.37)  *pr=0.09* | 3.09 (-7.57, 13.50)  *pr=0.29* | 1.84 (-1.92, 7.01)  *pr=0.13* | 0.04 (-0.03, 0.10)  *pr=0.14* |
| FJ:T – MA:T | 0.40 (0.15, 0.68)  *pr=0.00* | 7.45 (3.41, 11.98)  *pr=0.00* | 9.51 (-2.93, 18.65)  *pr=0.07* | 7.26 (3.06, 11.63)  *pr=0.00* | 0.10 (-0.03, 0.10)  *pr=0.01* |
| FA:T – MJ:T | 0.69 (0.44, 0.98)  *pr=0.00* | 13.82 (8.58, 17.40)  *pr=0.00* | 16.54 (3.19, 24.22)  *pr=0.01* | 12.25 (7.97, 16.94)  *pr=0.00* | 0.13 (0.07, 0.21)  *pr=0.00* |
| FA:T – MA:T | 0.48 (0.22, 0.75)  *pr=0.00* | 8.40 (3.53, 12.49)  *pr=0.00* | 13.96 (-1.61, 19.58)  *pr=0.05* | 8.52 (3.83, 12.58)  *pr=0.00* | 0.10 (0.02, 0.16)  *pr=0.00* |
| **MJ:T – MA:T** | -0.24 (-0.47, 0.06)  *pr=0.05* | -5.31 (-8.92, 0.02)  *pr=0.02* | -4.04 (-14.10, 6.34)  *pr=0.16* | -5.81 (-9.19, -0.28)  *pr=0.01* | -0.04 (-0.12, 0.02)  *pr=0.08* |

# Table S6. Off-territory model comparison with and without random slopes (IxE). See Table S5 for pairwise comparison proportion overlap (pr) values. See Figure S10 for DHARMa output.

|  | GLMM (as presented in main text) | GLMM + IxE |
| --- | --- | --- |
| *Fixed effects* | *β*  *(95% CrI)*  *Proportion overlap* | *β*  *(95% CrI)*  *Proportion overlap* |
| Female – Juvenile | -1.99  (-4.74, -0.15) | -3.03  (-4.80, 0.16)  *pr=0.04* |
| Female – Adult | -2.84  (-4.85, 0.17)  *pr=0.04* | -2.15  (-4.38, 0.64)  *pr=0.06* |
| Male – Juvenile | -2.26  (-4.55, 0.11)  *pr=0.03* | -1.41  (-4.09, 0.91)  *pr=0.08* |
| Male – Adult | -4.40  (-6.26, -1.37) | -3.89  (-6.13, -1.19) |
| Female – Juvenile : Temperature | -0.57  (-0.92, 0.01)  *pr=0.03* | -1.02  (-1.69, -0.14) |
| Female – Adult : Temperature | -0.78  (-1.28, -0.23) | -1.13  (-2.11, -0.43) |
| Male – Juvenile : Temperature | 0.23  (-0.33, 0.57)  *pr=0.28* | -0.24  (-1.18, 0.47)  *pr=0.17* |
| Male – Adult : Temperature | 0.02  (-0.41, 0.60)  *pr=0.33* | -0.35  (-1.30, 0.70)  *pr=0.24* |
| *Random Effects* | *σ*  *(95% CrI)* | *σ*  *(95% CrI)* |
| Bird ID N=138 | 4.43  (3.76, 5.72) | 0.64  (0.32, 4.86) |
| Core Feeder ID N=7 | 11.70  (2.52, 24.09) | 12.68  (3.18, 31.22) |
| Residual N=4969 | 1  (--) | 1  (--) |
| *Repeatability* | *r*  *(95% CrI)* | *r*  *(95% CrI)* |
| Bird ID N=138 | 0.57  (0.53, 0.63) | 0.18  (0.11, 0.60) |

# Table S7. Effect size contrasts for off-territory models with and without random slopes (IxE). Bolded pr-values are those that have a different pr-value strength of support in the IxE model. However, it is important to note that these differences do not change our interpretation of our results. The effects contrasts show that in the IxE model, the strength of the negative relationship between temperature and off-territory use differs between juvenile females and males; however, as discussed in the main text, each Age-Sex category still has a significant negative relationship. See Table S6 for model outputs. FJ = female, juvenile; FA = female, adult; MJ = male, juvenile; MA = male, adult; T = temperature interaction.

|  | GLMM (as presented in main text) | GLMM + IxE |
| --- | --- | --- |
| *Contrast* | *β (95% CrI)*  *Proportion overlap* | *β (95% CrI)*  *Proportion overlap* |
| FA – FJ | -0.02 (-1.19, 1.76)  *pr=0.51* | -0.03 (-1.08, 1.44)  *pr=0.59* |
| MJ – FJ | -1.51 (-2.86, 0.14)  *pr=0.03* | -1.34 (-2.69, 0.30)  *pr=0.05* |
| MJ – FA | 0.32 (-1.26, 1.45)  *pr=0.43* | 0.34 (-0.88, 1.80)  *pr=0.28* |
| MA – FJ | -0.36 (-1.26, 1.45)  *pr=0.54* | -0.04 (-1.08, 1.53)  *pr=0.63* |
| MA – FA | -1.27 (-2.99, 0.05)  *pr=0.03* | -1.02 (-2.93, 0.06)  *pr=0.03* |
| MA – MJ | -1.54 (-3.12, -0.23)  *pr=0.02* | -1.91 (-3.02, -0.27)  *pr=0.01* |
| FJ:T – FA:T | 0.34 (-0.47, 0.94)  *pr=0.24* | 0.60 (-0.86, 1.52)  *pr=0.32* |
| FJ:T – MJ:T | -0.61 (-1.26, 0.14)  *pr=0.05* | -0.68 (-1.82, 0.69)  ***pr=0.20*** |
| FJ:T – MA:T | -0.59 (-1.24, 0.01)  *pr=0.03* | -0.51 (-1.72, 0.48)  ***pr=0.18*** |
| FA:T – MJ:T | -0.77 (-1.62, -0.22)  *pr=0.01* | -1.07 (-1.94, 0.36)  *pr=0.08* |
| FA:T – MA:T | -1.03 (-1.62, -0.16)  *pr=0.01* | -0.61 (-2.13, 0.54)  *pr=0.10* |
| MJ:T – MA:T | -0.08 (-0.61, 0.75)  *pr=0.51* | -0.18 (-1.30, 1.23)  *pr=0.48* |

# Table S8. Rarefication results for GLMM models of off-territory use (1 = off territory feeders used, 0 = no off-territory feeders used) after removal of a single feeder (and all birds for which that feeder was the core feeder). Models were constructed with binomial error distribution. Proportion overlap (pr) values are provided for effect sizes with CrIs that overlap zero to show the proportion of estimates which overlap zero. See Table S9 below for proportion overlap (pr) of pairwise contrasts.

|  | Feeder02 | Feeder04 | Feeder09 | Feeder10 | Feeder11 | Feeder12 | Feeder16 |
| --- | --- | --- | --- | --- | --- | --- | --- |
| *Fixed Effects* | *β (95% CrI)*  *p-value* | *β (95% CrI)*  *p-value* | *β (95% CrI)*  *p-value* | *β (95% CrI)*  *p-value* | *β (95% CrI)*  *p-value* | *β (95% CrI)*  *p-value* | *β (95% CrI)*  *p-value* |
| Female - Juvenile^1^ | -1.90  (-3.91, 0.39)  *pr=0.07* | -2.45  (-4.93, 0.29)  *pr=0.05* | -3.20  (-5.87, -1.11) | -2.82  (-4.89, 0.46)  *pr=0.04* | -3.19  (-5.44, -0.21) | -2.18  (-4.43, 0.77)  *pr=0.07* | -1.61  (-3.90, 0.31)  *pr=0.05* |
| Female - Adult^1^ | -1.95  (-4.03, 0.72)  *pr=0.08* | -2.80  (-5.14, 0.39)  *pr=0.06* | -3.42  (-6.14, -1.06) | -1.81  (-4.60, 1.05)  *pr=0.08* | -3.83  (-5.43, 0.04)  *pr=0.02* | -2.60  (-4.87, 0.57)  *pr=0.05* | -1.96  (-4.00, 0.50)  *pr=0.10* |
| Male - Juvenile^1^ | -1.50  (-4.30, 0.35)  *pr=0.05* | -2.06  (-4.16, 1.15)  *pr=0.15* | -3.29  (-6.20, -1.25) | -2.28  (-4.88, 0.53)  *pr=0.07* | -2.56  (-5.43, 0.04)  *pr=0.03* | -1.98  (-4.57, 0.68)  *pr=0.07* | -2.03  (-3.92, 0.46)  *pr=0.06* |
| Male - Adult^1^ | -3.04  (-5.68, -1.02) | -3.32  (-6.46, -0.51) | -5.35  (-7.97, -2.90) | -3.55  (-6.13, -0.73) | -4.77  (-7.33, -1.68) | -3.40  (-6.28, -0.88) | -3.07  (-5.30, -0.76) |
| Female - Juvenile : Temp^2^ | -0.63  (-0.96, -0.03) | -0.82  (-1.45, -0.41) | 0.00  (-0.59, 0.41)  *pr=0.58* | -0.30  (-0.86, 0.15)  *pr=0.08* | -0.48  (-0.94, 0.06)  *pr=0.03* | -0.57  (-1.13, -0.18) | -0.45  (-0.92, -0.05) |
| Female - Adult : Temp^2^ | -0.82  (-1.43, -0.40) | -0.99  (-1.49, -0.26) | -0.30  (-0.77, 0.51)  *pr=0.27* | -0.70  (-1.18, -0.15) | -0.68  (-1.39, -0.30) | -0.64  (-1.33, -0.21) | -0.79  (-1.28, -0.21) |
| Male - Juvenile : Temp^2^ | 0.27  (-0.22, 0.74)  *pr=0.14* | -1.01  (-1.62, -0.49) | 0.66  (0.04, 1.11) | 0.11  (-0.29, 0.63)  *pr=0.24* | 0.52  (-0.02, 1.04)  *pr=0.03* | 0.11  (-0.30, 0.61)  *pr=0.25* | 0.16  (-0.29, 0.64)  *pr=0.27* |
| Male - Adult : Temp^2^ | 0.08  (-0.42, 0.64)  *pr=0.36* | -0.72  (-1.46, -0.10) | 1.14  (0.35, 1.66) | 0.11  (-0.41, 0.62)  *pr=0.34* | 0.05  (-0.54, 0.81)  *pr=0.35* | 0.05  (-0.46, 0.58)  *pr=0.41* | -0.01  (-0.38, 0.68)  *pr=0.66* |
| *Random Effects* | *σ (95% CrI)* | *σ (95% CrI)* | *σ (95% CrI)* | *σ (95% CrI)* | *σ (95% CrI)* | *σ (95% CrI)* | *σ (95% CrI)* |
| Bird ID | 3.62  (2.76, 4.45) | 4.87  (3.71, 6.17) | 5.83  (4.53, 7.08) | 4.62  (3.70, 5.86) | 5.33  (4.23, 6.89) | 4.48  (3.66, 5.74) | 4.63  (3.62, 5.68) |
| Core Feeder | 12.14  (2.10, 23.48) | 15.39  (3.06, 29.51) | 6.78  (1.97, 20.02) | 10.83  (3.55, 29.82) | 11.61  (3.26, 29.98) | 13.07  (2.72, 28.23) | 6.34  (2.02, 13.09) |
| Residual | 1 (--) | 1 (--) | 1 (--) | 1 (--) | 1 (--) | 1 (--) | 1 (--) |
| *Repeatability* | *r (95% CrI)* | *r (95% CrI)* | *r (95% CrI)* | *r (95% CrI)* | *r (95% CrI)* | *r (95% CrI)* | *r (95% CrI)* |
| Bird ID | 0.53  (0.47, 0.58) | 0.60  (0.53, 0.65) | 0.64  (0.58, 0.69) | 0.60  (0.53, 0.64) | 0.62  (0.57, 0.68) | 0.58  (0.53, 0.64) | 0.60  (0.53, 0.64) |

^1^Note, intercept values for Age-Sex categories are estimated at -17.1°C (the lowest temperature in our dataset)

^2^Temperature was standardized and left-zeroed prior to analysis, therefore estimate effect sizes are for 1 s.d. change in temperature (i.e., 5.74°C).

# Table S9. Proportion overlap (pr) values for pairwise contrasts of estimates for off-territory use rarefaction analyses presented in Table S8. Off-territory use (1 = off territory feeders used, 0 = no off-territory feeders used) GLMM models were constructed with binomial error distribution. Estimated differences are calculated by subtracting the two estimates in the “Contrasts” column as shown. FJ = female, juvenile; FA = female, adult; MJ = male, juvenile; MA = male, adult; T = temperature interaction.

|  | Feeder02 | Feeder04 | Feeder09 | Feeder10 | Feeder11 | Feeder12 | Feeder16 |
| --- | --- | --- | --- | --- | --- | --- | --- |
| *Contrasts* | *β (95% CrI)*  *p-value* | *β (95% CrI)*  *p-value* | *β (95% CrI)*  *p-value* | *β (95% CrI)*  *p-value* | *β (95% CrI)*  *p-value* | *β (95% CrI)*  *p-value* | *β (95% CrI)*  *p-value* |
| FA – FJ | 0.04 (-1.34, 1.39)  *pr=0.49* | 0.04 (-1.34, 1.39)  *pr=0.52* | -0.07 (-1.80, 1.57)  *pr=0.44* | -0.02 (-1.19, 1.96)  *pr=0.69* | -0.55 (-1.82, 1.49)  *pr=0.43* | -0.17 (-1.58, 1.29)  *pr=0.36* | 0.28 (-1.14, 1.67)  *pr=0.41* |
| MJ – FJ | -1.63 (-2.96, -0.02)  *pr=0.03* | -1.40 (-3.07, 0.43)  *pr=0.08* | -1.83 (-3.89, -0.46)  *pr=0.01* | -1.14 (-2.80, 0.36)  *pr=0.07* | -2.21 (-3.65, 0.31)  *pr=0.04* | -1.53 (-2.97, -0.06)  *pr=0.02* | -1.47 (-2.90, 0.14)  *pr=0.05* |
| MJ – FA | 0.00 (-1.66, 1.08)  *pr=0.40* | 0.97 (-0.77, 2.41)  *pr=0.18* | -0.26 (-1.77, 1.65)  *pr=0.46* | 0.03 (-1.10, 1.74)  *pr=0.37* | -0.06 (-1.77, 1.69)  *pr=0.52* | -0.10 (-1.40, 1.40)  *pr=0.53* | 0.43 (-1.45, 1.46)  *pr=0.46* |
| MA – FJ | -0.57 (-1.66, 1.33)  *pr=0.39* | 0.58 (-0.92, 2.58)  *pr=0.21* | 0.14 (-1.74, 1.67)  *pr=0.50* | 0.05 (-1.56, 1.23)  *pr=0.55* | 0.00 (-1.52, 1.90)  *pr=0.41* | 0.17 (-1.02, 1.96)  *pr=0.34* | 0.14 (-1.50, 1.55)  *pr=0.56* |
| MA – FA | -1.22 (-2.94, 0.07)  *pr=0.03* | -1.53 (-3.38, 0.42)  *pr=0.09* | -1.96 (-3.96, -0.28)  *pr=0.02* | -1.67 (-3.17, -0.04)  *pr=0.02* | -1.92 (-3.69, 0.08)  *pr=0.04* | -1.25 (-2.79, 0.23)  *pr=0.05* | -1.80 (-3.05, -0.01)  *pr=0.03* |
| MA – MJ | -1.10 (-2.68, 0.19)  *pr=0.05* | -1.95 (-4.00, -0.47)  *pr=0.01* | -1.56 (-3.95, -0.27)  *pr=0.02* | -1.26 (-2.90, 0.13)  *pr=0.03* | -1.91 (-3.69, 0.00)  *pr=0.02* | -1.76 (-3.08, -0.13)  *pr=0.01* | -0.87 (-2.70, 0.16)  *pr=0.03* |
| FJ:T – FA:T | 0.45 (-0.24, 1.13)  *pr=0.12* | -0.07 (-0.73, 0.77)  *pr=0.52* | 0.29 (-0.71, 0.89)  *pr=0.36* | 0.28 (-0.35, 1.04)  *pr=0.18* | 0.48 (-0.33, 1.10)  *pr=0.19* | 0.06 (-0.59, 0.83)  *pr=0.39* | 0.17 (-0.46, 0.94)  *pr=0.21* |
| FJ:T – MJ:T | -0.27 (-1.25, 0.16)  *pr=0.05* | -0.08 (-1.03, 0.69)  *pr=0.38* | -1.17 (-1.92, -0.26)  *pr=0.01* | -0.44 (-1.30, 0.20)  *pr=0.09* | -0.65 (-1.46, 0.27)  *pr=0.08* | -0.66 (-1.43, -0.04)  *pr=0.02* | -0.62 (-1.35, 0.03)  *pr=0.04* |
| FJ:T – MA:T | -0.80 (-1.40, -0.10)  *pr=0.01* | -0.80 (-1.40, -0.10)  *pr=0.35* | -0.51 (-1.41, 0.05)  *pr=0.04* | -0.49 (-1.21, 0.13)  *pr=0.05* | -0.91 (-1.74, -0.28)  *pr=0.00* | -0.82 (-1.40, -0.16)  *pr=0.01* | -0.57 (-1.26, 0.06)  *pr=0.03* |
| FA:T – MJ:T | -1.29 (-1.83, -0.52)  *pr=0.00* | 0.04 (-0.78, 0.89)  *pr=0.37* | -0.92 (-1.56, 0.10)  *pr=0.03* | -0.88 (-1.62, -0.19)  *pr=0.01* | -1.23 (-2.11, -0.56)  *pr=0.00* | -0.98 (-1.66, -0.22)  *pr=0.00* | -0.85 (-1.57, -0.20)  *pr=0.00* |
| FA:T – MA:T | -0.94 (-1.78, -0.26)  *pr=0.00* | -0.29(-0.98, 0.86)  *pr=0.35* | -1.33 (-2.05, -0.23)  *pr=0.01* | -0.75 (-1.59, -0.13)  *pr=0.01* | -0.77 (-1.76, -0.08)  *pr=0.02* | -0.94 (-1.66, -0.22)  *pr=0.02* | -1.03 (-1.72, -0.20)  *pr=0.01* |
| MJ:T – MA:T | 0.10 (-0.53, 0.88)  *pr=0.34* | 0.07 (-1.14, 0.51)  *pr=0.74* | -0.33 (-1.21, 0.51)  *pr=0.20* | -0.06 (-0.65, 0.70)  *pr=0.56* | 0.37 (-0.43, 1.26)  *pr=0.20* | 0.12 (-0.61, 0.77)  *pr=0.39* | 0.10 (-0.68, 0.75)  *pr=0.49* |

# Table S10. Rarefication results for LMM models of square root (daily feeder visits) after removal of a single feeder (and all birds for which that feeder was the core feeder). See Table S11 below for proportion overlap (pr) values of pairwise contrasts.

|  | Feeder02 | Feeder04 | Feeder09 | Feeder10 | Feeder11 | Feeder12 | Feeder16 |
| --- | --- | --- | --- | --- | --- | --- | --- |
| *Fixed Effects* | *β (95% CrI)* | *β (95% CrI)* | *β (95% CrI)* | *β (95% CrI)* | *β (95% CrI)* | *β (95% CrI)* | *β (95% CrI)* |
| Female - Juvenile^1^ | 8.54  (7.97, 9.29) | 8.67  (7.95, 9.03) | 8.60  (8.12, 9.20) | 8.58  (8.06, 9.23) | 8.79  (8.11, 9.29) | 8.57  (8.04, 9.10) | 8.66  (7.96, 9.21) |
| Female - Adult^1^ | 8.32  (7.65, 9.11) | 8.41  (7.65, 8.80) | 8.49  (7.88, 9.09) | 8.37  (7.70, 8.91) | 8.39  (7.81, 9.05) | 8.09  (7.66, 8.74) | 8.24  (7.73, 8.95) |
| Male - Juvenile^1^ | 9.61  (8.87, 10.28) | 9.58  (9.10, 10.29) | 9.56  (8.89, 10.10) | 9.65  (9.12, 10.31) | 9.58  (9.15, 10.43) | 9.62  (9.07, 10.17) | 9.60  (8.89, 10.13) |
| Male - Adult^1^ | 9.68  (8.76, 10.08) | 9.33  (8.93, 10.02) | 9.49  (8.92, 10.05) | 9.34  (8.84, 10.04) | 9.22  (8.63, 9.94) | 9.43  (8.75, 9.87) | 9.74  (8.99, 10.30) |
| Female - Juvenile : Temp^2^ | -1.07  (-1.33, -0.88) | -0.95  (-1.14, -0.74) | -1.17  (-1.38, -0.99) | -1.02  (-1.20, -0.82) | -1.11  (-1.34, -0.94) | -1.05  (-1.27, -0.88) | -1.07  (-1.26, -0.88) |
| Female - Adult : Temp^2^ | -0.67  (-0.90, -0.42) | -0.56  (-0.74, -0.32) | -0.91  (-1.12, -0.72) | -0.94  (-1.13, -0.72) | -0.83  (-1.04, -0.63) | -0.66  (-0.92, -0.51) | -0.86  (-1.04, -0.66) |
| Male - Juvenile : Temp^2^ | -1.41  (-1.65, -1.18) | -1.34  (-1.55, -1.13) | -1.54  (-1.76, -1.36) | -1.54  (-1.71, -1.34) | -1.66  (-1.83, -1.40) | -1.49  (-1.68, -1.30) | -1.52  (-1.67, -1.27) |
| Male - Adult : Temp^2^ | -1.15  (-1.37, -0.94) | -1.18  (-1.34, -0.92) | -1.38  (-1.60, -1.20) | -1.39  (-1.59, -1.20) | -1.13  (-1.34, -0.89) | -1.41  (-1.62, -1.22) | -1.08  (-1.37, -0.96) |
| *Random Effects* | *σ (95% CrI)* | *σ (95% CrI)* | *σ (95% CrI)* | *σ (95% CrI)* | *σ (95% CrI)* | *σ (95% CrI)* | *σ (95% CrI)* |
| Bird ID | 2.48  (2.08, 2.80) | 2.01  (1.83, 2.45) | 2.10  (1.88, 2.41) | 2.04  (1.87, 2.33) | 1.94  (1.71, 2.24) | 1.87  (1.60, 2.18) | 2.00  (1.81, 2.31) |
| Core Feeder | 0.08  (0.03, 0.15) | 0.00  (0.00, 0.00) | 0.05  (0.02, 0.09) | 0.09  (0.04, 0.14) | 0.11  (0.06, 0.19) | 0.04  (0.02, 0.09) | 0.08  (0.04, 0.26) |
| Residual | 2.13  (2.01, 2.20) | 1.83  (1.73, 1.88) | 1.85  (1.78, 1.92) | 1.80  (1.73, 1.88) | 1.94  (1.85, 2.01) | 1.87  (1.80, 1.95) | 1.72  (1.66, 1.81) |
| *Repeatability* | *r (95% CrI)* | *r (95% CrI)* | *r (95% CrI)* | *r (95% CrI)* | *r (95% CrI)* | *r (95% CrI)* | *r (95% CrI)* |
| Bird ID | 0.51  (0.42, 0.59) | 0.53  (0.46, 0.59) | 0.52  (0.44, 0.58) | 0.51  (0.43, 0.58) | 0.47  (0.38, 0.55) | 0.48  (0.41, 0.55) | 0.51  (0.42, 0.59) |

^1^Note, intercept values for Age-Sex categories are estimated at -17.1°C (the lowest temperature in our dataset)

^2^Temperature was standardized and left-zeroed prior to analysis, therefore estimate effect sizes are for 1 s.d. change in temperature (i.e., 5.74°C).

# Table S11. Proportion overlap (pr) values for pairwise contrasts of estimates for feeder visit rate rarefaction analyses presented in Table S10. Estimated differences are calculated by subtracting the two estimates in the “Contrasts” column as shown. FJ = female, juvenile; FA = female, adult; MJ = male, juvenile; MA = male, adult; T = temperature interaction.

|  | Feeder02 | Feeder04 | Feeder09 | Feeder10 | Feeder11 | Feeder12 | Feeder16 |
| --- | --- | --- | --- | --- | --- | --- | --- |
| *Contrasts* | *β (95% CrI)*  *p-value* | *β (95% CrI)*  *p-value* | *β (95% CrI)*  *p-value* | *β (95% CrI)*  *p-value* | *β (95% CrI)*  *p-value* | *β (95% CrI)*  *p-value* | *β (95% CrI)*  *p-value* |
| FA – FJ | -0.20 (-1.04, 0.69)  *pr=0.32* | -0.11 (-1.02, 0.56)  *pr=0.25* | -0.05 (-0.89, 0.57)  *pr=0.36* | -0.30 (-1.11, 0.36)  *pr=0.17* | -0.23 (-0.97, 0.47)  *pr=0.25* | -0.17 (-1.58, 1.29)  *pr=0.36* | -0.34 (-1.08, 0.42)  *pr=0.20* |
| MJ – FJ | 0.66 (-0.09, 1.58)  *pr=0.04* | 0.79 (0.13, 1.63)  *pr=0.01* | 0.87 (0.13, 1.54)  *pr=0.01* | 0.61 (0.05, 1.43)  *pr=0.02* | 0.62 (-0.12, 1.40)  *pr=0.07* | -1.53 (-2.97, -0.06)  *pr=0.02* | 0.93 (0.31, 1.79)  *pr=0.00* |
| MJ – FA | 1.00 (0.10, 1.83)  *pr=0.02* | 1.26 (0.33, 1.97)  *pr=0.00* | 0.82 (0.27, 1.76)  *pr=0.00* | 0.99 (0.24, 1.70)  *pr=0.00* | 1.15 (0.33, 1.86)  *pr=0.00* | -0.10 (-1.40, 1.40)  *pr=0.53* | 0.85 (0.19, 1.71)  *pr=0.01* |
| MA – FJ | 1.21 (0.32, 2.12)  *pr=0.01* | 1.50 (0.54, 2.17)  *pr=0.00* | 1.27 (0.37, 1.97)  *pr=0.00* | 1.28 (0.56, 2.11)  *pr=0.00* | 1.20 (0.59, 2.07)  *pr=0.00* | 0.17 (-1.02, 1.96)  *pr=0.34* | 1.20 (0.43, 1.95)  *pr=0.00* |
| MA – FA | 0.84 (0.19, 1.85)  *pr=0.01* | 1.31 (0.36, 1.98)  *pr=0.00* | 0.93 (0.24, 1.71)  *pr=0.00* | 1.08 (0.38, 1.90)  *pr=0.00* | 0.90 (0.09, 1.55)  *pr=0.02* | -1.25 (-2.79, 0.23)  *pr=0.05* | 1.20 (0.59, 2.17)  *pr=0.00* |
| MA – MJ | -0.44 (-1.03, 0.62)  *pr=0.35* | -0.39 (-1.10, 0.55)  *pr=0.30* | -0.06 (-0.84, 0.65)  *pr=0.35* | -0.13 (-0.95, 0.47)  *pr=0.27* | -0.35 (-1.25, 0.26)  *pr=0.09* | -1.76 (-3.08, -0.13)  *pr=0.01* | 0.15 (-0.51, 0.95)  *pr=0.32* |
| FJ:T – FA:T | -0.45 (-0.73, -0.11)  *pr=0.01* | -0.35 (-0.70, -0.10)  *pr=0.00* | -0.21 (-0.50, 0.06)  *pr=0.05* | -0.17 (-0.34, 0.19)  *pr=0.33* | -0.30 (-0.64, -0.05)  *pr=0.01* | 0.06 (-0.59, 0.83)  *pr=0.39* | -0.27 (-0.49, 0.04)  *pr=0.07* |
| FJ:T – MJ:T | 0.01 (-0.20, 0.41)  *pr=0.32* | 0.15 (-0.07, 0.52)  *pr=0.09* | 0.20 (-0.05, 0.45)  *pr=0.06* | 0.40 (0.15, 0.68)  *pr=0.00* | -0.04 (-0.32, 0.26)  *pr=0.48* | -0.66 (-1.43, -0.04)  *pr=0.02* | 0.06 (-0.19, 0.40)  *pr=0.24* |
| FJ:T – MA:T | 0.28 (0.01, 0.63)  *pr=0.02* | 0.46 (0.11, 0.70)  *pr=0.00* | 0.48 (0.20, 0.73)  *pr=0.00* | 0.48 (0.24, 0.80)  *pr=0.00* | 0.45 (0.12, 0.70)  *pr=0.00* | -0.82 (-1.40, -0.16)  *pr=0.01* | 0.42 (0.16, 0.68)  *pr=0.00* |
| FA:T – MJ:T | 0.62 (0.42, 1.08)  *pr=0.00* | 0.94 (0.50, 1.12)  *pr=0.00* | 0.77 (0.41, 0.99)  *pr=0.00* | 0.62 (0.35, 0.89)  *pr=0.00* | 0.72 (0.48, 1.09)  *pr=0.00* | -0.98 (-1.66, -0.22)  *pr=0.00* | 0.58 (0.32, 0.88)  *pr=0.00* |
| FA:T – MA:T | 0.51 (0.17, 0.81)  *pr=0.00* | 0.65 (0.28, 0.91)  *pr=0.00* | 0.45 (0.19, 0.73)  *pr=0.00* | 0.50 (0.20, 0.75)  *pr=0.00* | 0.39 (0.01, 0.61)  *pr=0.02* | -0.94 (-1.67, -0.15)  *pr=0.02* | 0.33 (0.00, 0.61)  *pr=0.02* |
| MJ:T – MA:T | -0.26 (-0.55, 0.07)  *pr=0.07* | -0.16 (-0.51, 0.11)  *pr=0.07* | -0.28 (-0.50, 0.04)  *pr=0.05* | -0.12 (-0.40, 0.16)  *pr=0.19* | -0.40 (-0.75, -0.14)  *pr=0.00* | 0.12 (-0.61, 0.77)  *pr=0.39* | -0.29 (-0.60, -0.03)  *pr=0.02* |


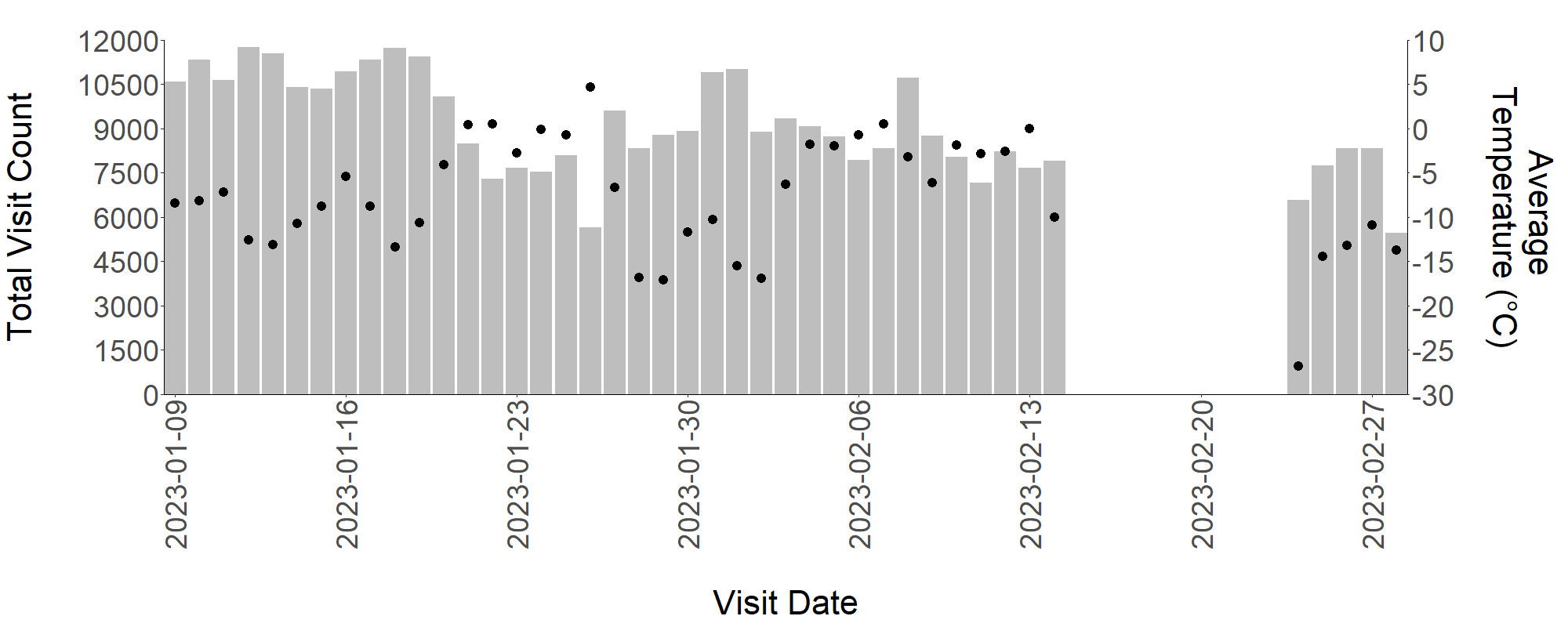


# Figure S1. Distribution of total visit counts summed by day (grey bars) and average daily temperature in °C (black dots) across the study period. There was a circuit board failure between February 18^th^ and 23^rd^, 2023, resulting in missing data for this period.


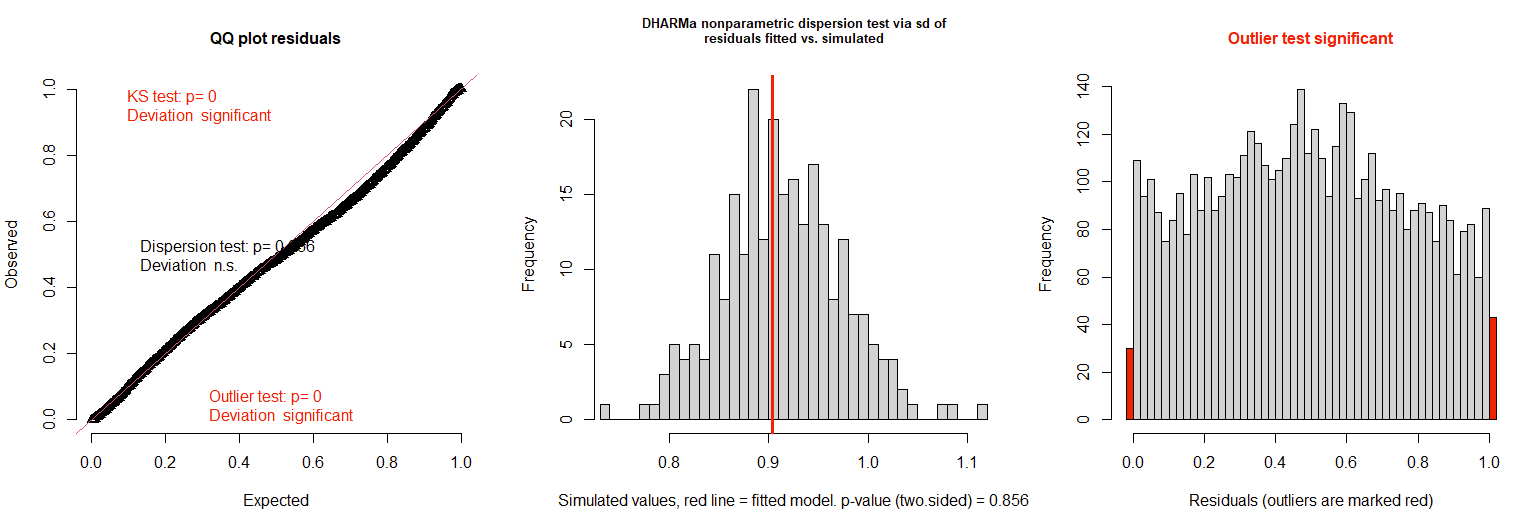


# **Figure S2**. DHARMa results for the square-root LMM for daily feeder visits (presented in main text). Note that visual inspection of the plots shows: 1) left plot – minimal deviation in the QQ plot away from the red 1:1 line, 2) center plot – no significant deviation in the dispersion of residuals as indicated by the red vertical line falling near the mean of the residuals, and 3) right plot - evidence of outliers in residuals. Importantly, outlier residuals are always under-represented. Underrepresentation of outliers is generally not problematic as it reduces power and leads to conservative conclusions.


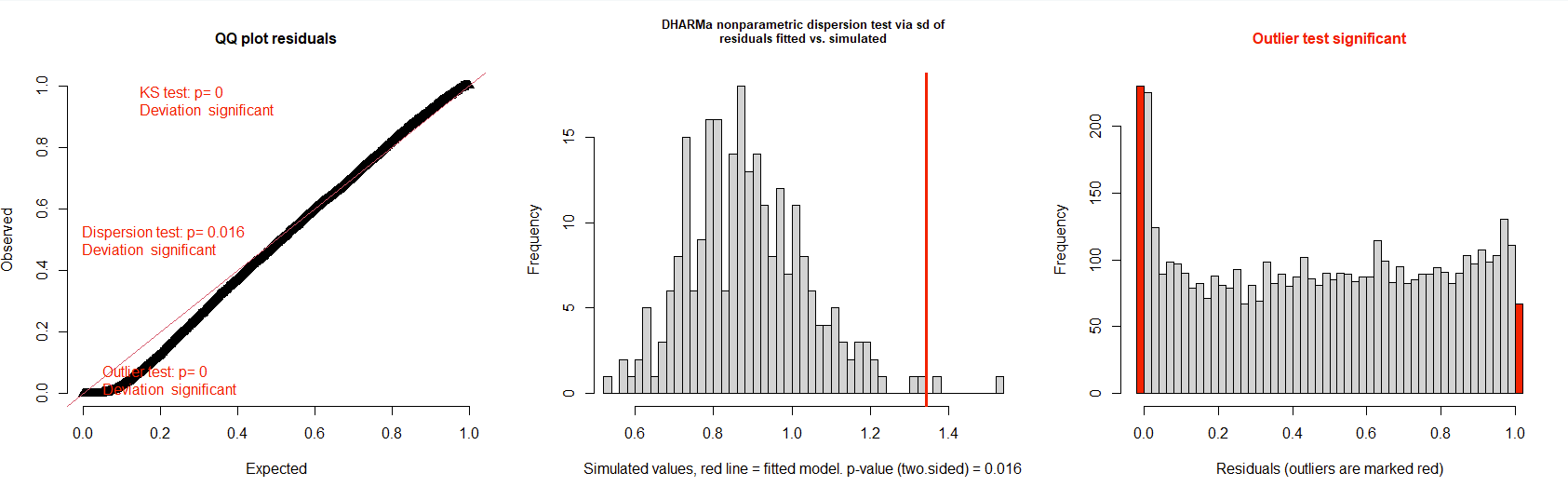


# Figure S3. DHARMa results for a non-transformed LMM for daily feeder visits. Note that visual inspection of the plots shows: 1) left plot - deviation in the QQ plot away from the red 1:1 line, 2) center plot - significant deviation in the dispersion of residuals as indicated by the red vertical line not falling near the mean of the residuals, and 3) right plot - evidence of outliers in residuals. Importantly, small residuals are over-represented, which can lead to underestimation of errors and therefore, overconfidence in estimate effects. Larger residuals are under-represented, which is generally not problematic as it reduces power and leads to conservative conclusions.

**
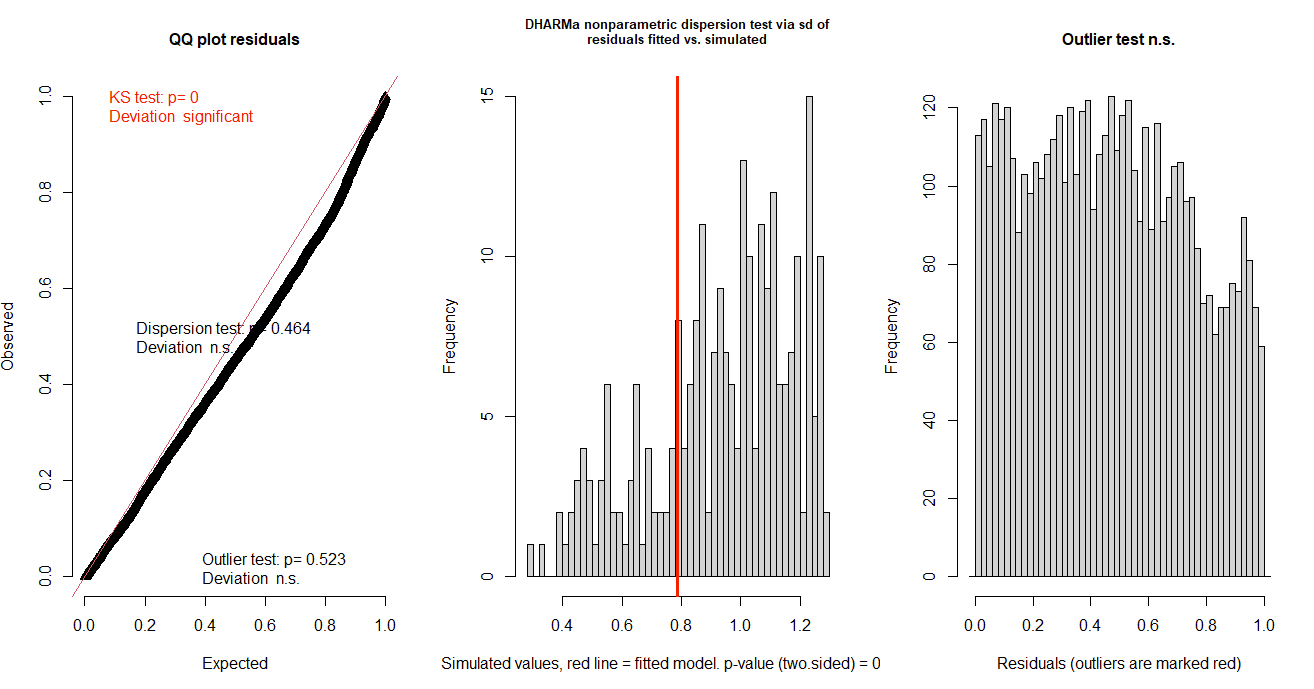
**

Figure S4. DHARMa results for including random slopes (IxE) in the square root transformed LMM for daily feeder visits. Note that visual inspection of the plots shows: 1) left plot - deviation in the QQ plot away from the red 1:1 line, 2) center plot – no significant deviation in the dispersion of residuals as indicated by the red vertical line falling near the mean of the residuals, and 3) right plot - no evidence of outliers in residuals.

**
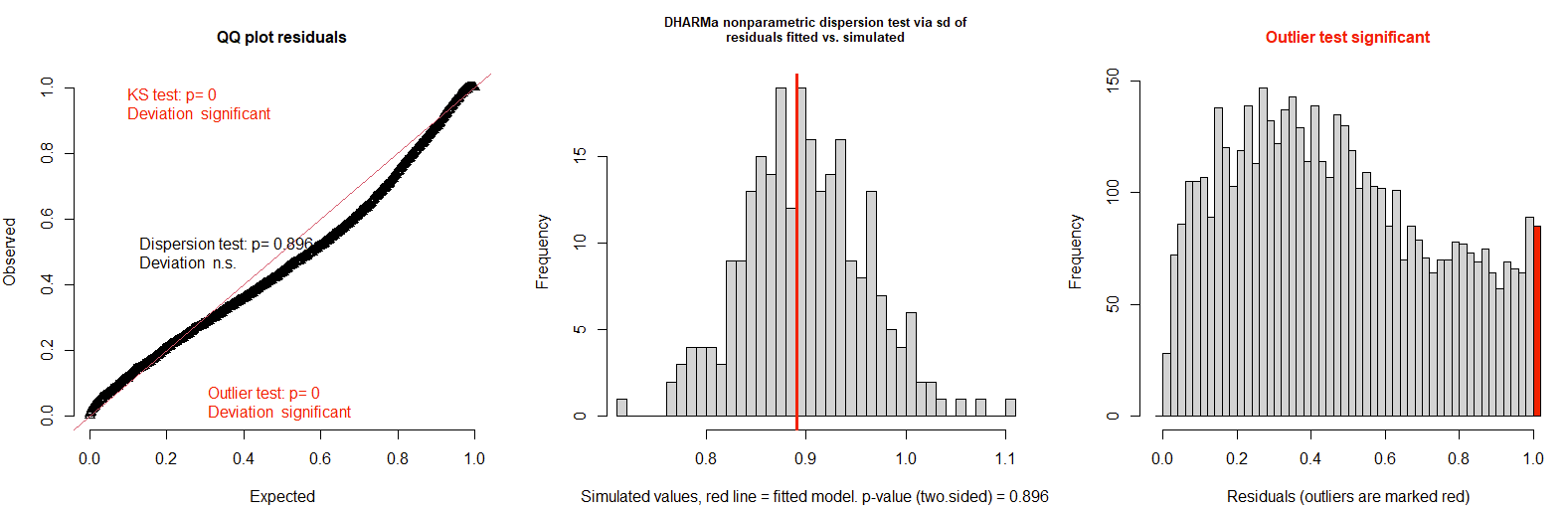
**

# Figure S5. DHARMa results for a quadratic LMM for daily feeder visits. Note that visual inspection of the plots shows: 1) left plot - deviation in the QQ plot away from the red 1:1 line, 2) center plot – no significant deviation in the dispersion of residuals as indicated by the red vertical line falling near the mean of the residuals, and 3) right plot - evidence of outliers in residuals. Importantly, large residuals are over-represented (indicated by red bar on far right of histogram), which can lead to underestimation of errors and therefore, overconfidence in estimate effects.


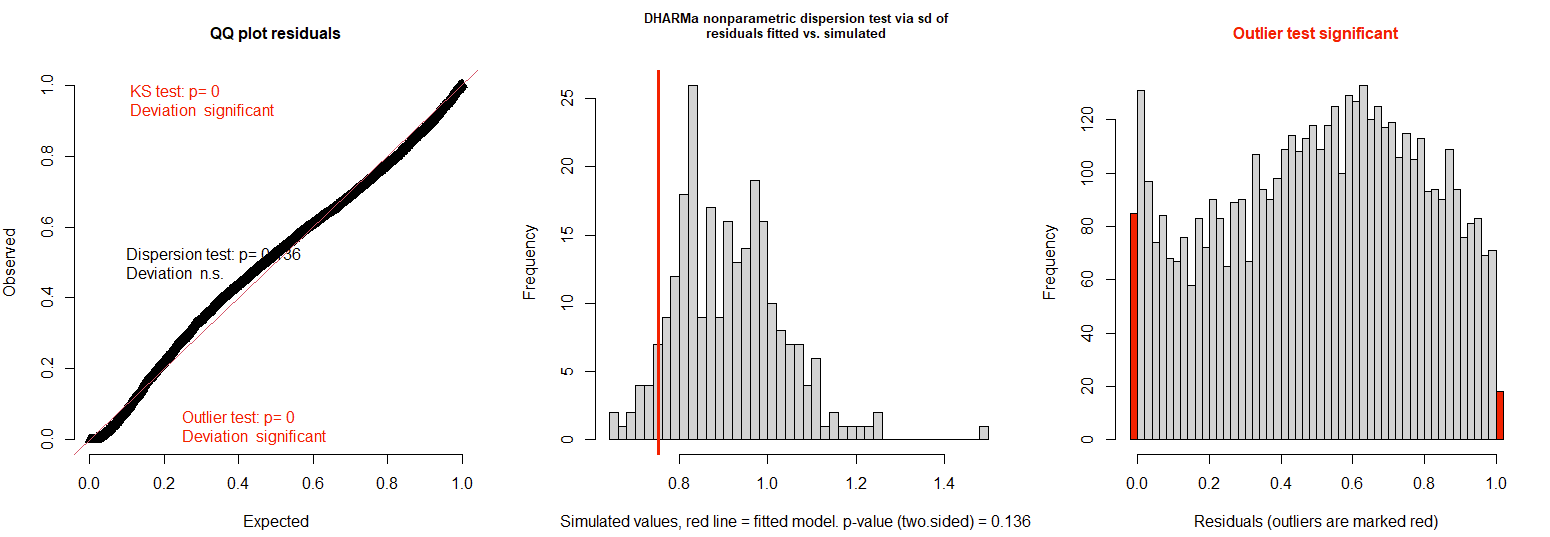


# Figure S6. DHARMa results for a negative binomial GLMM for daily feeder visits. Note that visual inspection of the plots shows: 1) left plot – small deviation in the QQ plot away from the red 1:1 line, 2) center plot - significant deviation in the dispersion of residuals as indicated by the red vertical line not falling near the mean of the residuals, and 3) right plot - evidence of outliers in residuals. Importantly, small residuals outliers are over-represented, which can lead to underestimation of errors and therefore, overconfidence in estimate effects. Larger residuals outliers are under-represented, which is generally not problematic as it reduces power and leads to conservative conclusons.


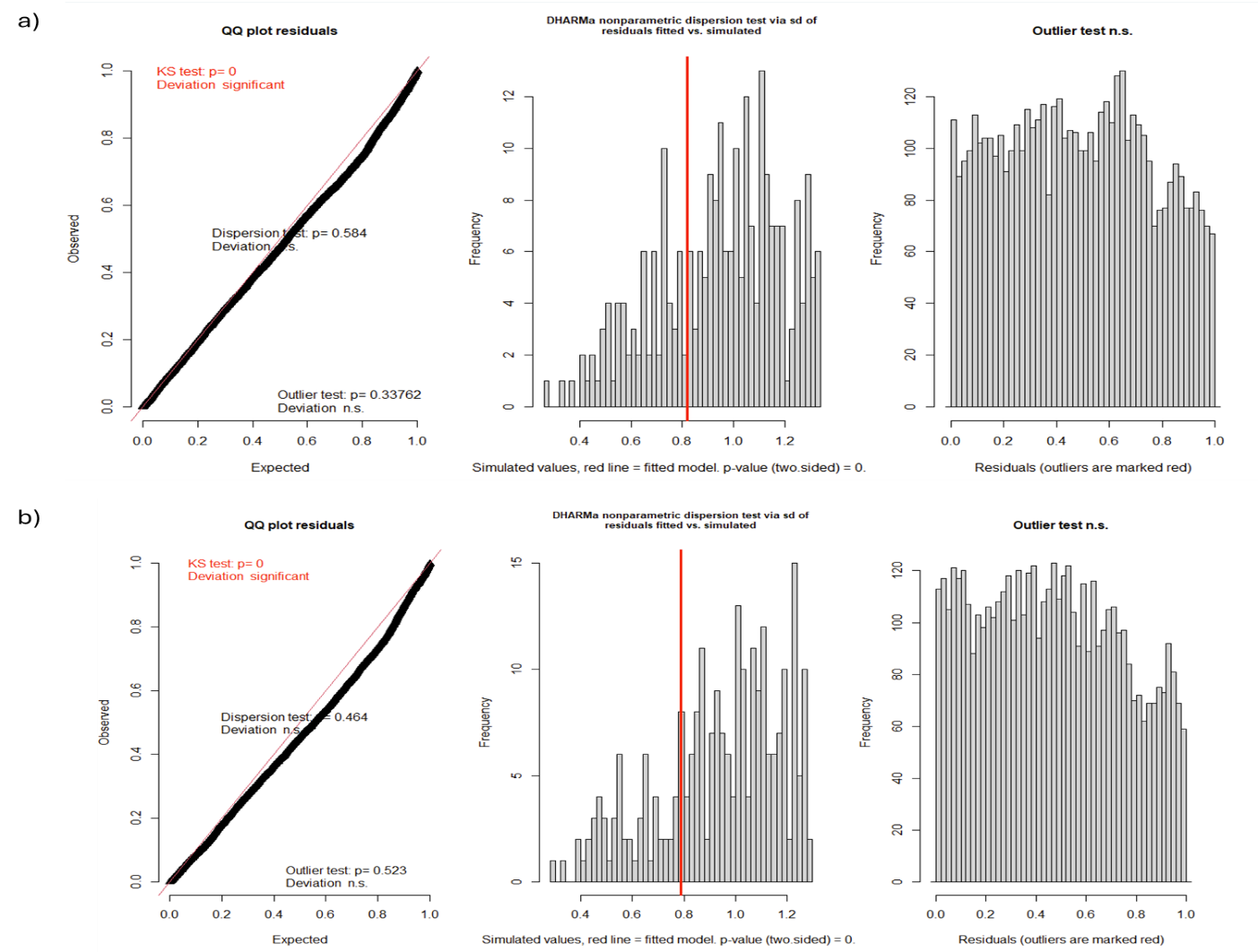


# Figure S7. DHARMa results for the off-territory model (a) excluding (as presented in the main text) and (b) including IxE. Note that visual inspection of the plots shows: 1) left plot - deviation in the QQ plot away from the red 1:1 line for both models, but more marked deviation whenincluding IxE (lower panel), 2) center plot – no significant deviation in the dispersion of residuals as indicated by the red vertical line falling near the mean of the residuals, and 3) right plot – no evidence of outliers in residuals.

# Supplementary References

Hartig F. DHARMa: Residual Diagnostics for Hierarchical (Multi-Level / Mixed) Regression Models; 2022.

R Development Core Team. R: a language and environment for statistical computing. 4.0.3. Vienna, Austria: See <http://www.R-project.org>; 2020.

R Studio Team. RStudio: integrated development for R. Boston, MA: RStudio, PBC; 2020.

Smith SM. The black-capped chickadee: behavioral ecology and natural history. Ithaca, NY: Cornell 466 University Press; 1992.

Sridharan S. Investigating the value of incorporating behavioural measures in a discriminant function developed for sex assignment in black-capped chickadees (*Poecile atricapillus*) [thesis]. Edmonton: University of Alberta; 2021.
